# Proteasome inhibition as a therapeutic target for the fungal pathogen *Cryptococcus neoformans*

**DOI:** 10.1128/spectrum.01904-23

**Published:** 2023-09-26

**Authors:** Mélissa Caza, Daniel Assis Santos, Elizabeth Burden, Anna Brisland, Guanggan Hu, James W. Kronstad

**Affiliations:** 1 Department of Microbiology and Immunology, Michael Smith Laboratories, University of British Columbia, Vancouver, British Columbia, Canada; 2 Department of Microbiology, Universidade Federal de Minas Gerais, Belo Horizonte, Minas Gerais, Brazil; Institut Pasteur, Paris, France

**Keywords:** bortezomib, chemical genetic screen, fungal pathogenesis, HIV/AIDS

## Abstract

The current therapeutic challenges for treating fungal diseases demand new approaches and new drugs. A promising strategy involves combination therapy with agents of distinct mechanisms of action to increase fungicidal activity and limit the impact of mutations leading to resistance. In this study, we evaluated the antifungal potential of bortezomib by examining the inhibition of proteasome activity, cell proliferation, and capsule production by *Cryptococcus neoformans*, the causative agent of fungal meningoencephalitis. Chemical genetic screens with collections of deletion mutants identified potential druggable targets for combination therapy with bortezomib. In vitro assays of combinations of bortezomib with flucytosine, chlorpromazine, bafilomycin A1, copper sulfate, or hydroxyurea revealed antifungal effects against *C. neoformans*. Furthermore, combination treatment with bortezomib and flucytosine in a murine inhalation model of cryptococcosis resulted in the improvement of neurological functions and reduced fungal replication and dissemination, leading to a delay in disease progression. This study therefore highlights the utility of chemical genetic screens to identify new therapeutic approaches as well as the antifungal potential of proteasome inhibition.

**IMPORTANCE:**

Fungal diseases of humans are difficult to treat, and there is a clear need for additional antifungal drugs, better diagnostics, effective vaccines, and new approaches to deal with emerging drug resistance. Fungi are challenging to control because they share many common biochemical functions with their mammalian hosts and it is therefore difficult to identify fungal-specific targets for drug development. One approach is to employ existing antifungal drugs in combination with agents that target common cellular processes at levels that are (ideally) not toxic for the host. We pursued this approach in this study by examining the potential of the clinically approved proteasome inhibitor bortezomib to influence the proliferation and virulence of *Cryptococcus neoformans*. We found that the combination of bortezomib with the anti-cryptococcal drug flucytosine improved the survival of infected mice, thus demonstrating the potential of this strategy for antifungal therapy.

## INTRODUCTION

Relatively few drugs are available to treat life-threatening fungal diseases in humans, with the available therapeutic options limited by toxicity, accessibility, and resistance (1 – 4). In this context, there is a pressing need to identify strategies to enhance the efficacy of existing antifungal drugs and to block the emergence of resistance. The therapeutic challenges in treating fungal diseases are clearly illustrated by the pathogen *Cryptococcus neoformans* (2, 3). This opportunistic fungus causes meningoencephalitis in immunocompromised individuals, such as those suffering from HIV/AIDS, with an estimated occurrence of 220,000 cases and 181,100 deaths annually (5). *C. neoformans* is refractory to treatment with echinocandins, thereby limiting therapeutic options to amphotericin B, flucytosine, and fluconazole (2 – 4). Resistance to these drugs in clinical isolates is an emerging problem and additional approaches to drug development, including targeting factors that contribute to virulence, are needed to establish new therapeutic avenues (1, 3, 4, 6 – 9). A promising approach is to employ combination therapy with two agents with distinct mechanisms of action to increase fungicidal activity, reduce the pathogen population size, and limit the impact of mutations leading to resistance (4). Additionally, combination therapy can potentially allow lower doses of each individual drug, and reduce treatment duration and host toxicity (3, 4).

The features that contribute to the virulence of *C. neoformans* are its ability to survive at 37°C, the production of melanin in the cell wall, the secretion of various enzymes including urease, and the elaboration of a polysaccharide capsule at the cell surface (10). The capsule enables evasion of the immune response by interfering with phagocytosis (11), blocking antigen presentation to T cells (12, 13), suppressing inflammatory cytokine production (11 – 14), blocking initiation of the classical complement pathway, and tempering deposition of complement component 3 (C3) on the cell surface (15, 16). Furthermore, the capsule affects the potency of antifungal drugs resulting in higher minimal inhibitory concentrations (17). *C. neoformans* mutants with capsule defects are unable to cause disease in mice, indicating that capsule production is important for virulence (18, 19). Capsule elaboration is regulated in part by the cAMP-PKA pathway, and a proteomic study of Pka1 targets recently unveiled a link between capsule elaboration and proteasome activity (18, 20). In particular, exposing *C. neoformans* to the proteasome inhibitor bortezomib causes a reduction in capsule formation, although the specific underlying mechanism is unknown (20).

Given the influence of bortezomib on capsule formation, proteasome inhibition may be a promising target for the treatment of cryptococcosis. The 26S proteasome machinery is highly conserved in eukaryotic cells, and ancestral forms are found in archaea and some bacteria (21). The 26S proteasome is a large multi-catalytic ATP-dependent protease complex, composed of nearly 40 different protein subunits, that degrades cytosolic, nuclear, and membrane proteins (22). It consists of a 20S core particle (CP), and a 19S regulatory particle (RP) that associates at one or both ends of the proteasome core particle (23). The catalytic activities of the core particle are found in binding pockets of three different β-subunits conferring chymotrypsin-like (β5), trypsin-like (β2), and caspase-like (β1) peptide cleavage activities. Proteasome inhibitors bind specifically to the β subunits to hinder their catalytic activities (24, 25). For example, bortezomib impedes the chymotrypsin-like activity of the β5 subunit (25 – 28). Given that proteasome function is essential for protein homeostasis and its activity influences the regulation of many cellular processes, inhibition of the proteasome catalytic activities by specific therapeutic agents has proven effective in cancer treatments, and the proteasome has been investigated as a therapeutic target for treating infectious diseases (26, 27).

In this study, we evaluated the antifungal potential of bortezomib by examining the inhibition of cryptococcal proteasome activity, cell proliferation, and capsule production. Chemical genetic screens with a collection of deletion mutants identified categories of biological functions as potential druggable targets for combination therapy with bortezomib. In vitro assays of combinations of bortezomib with flucytosine, chlorpromazine, bafilomycin A1, copper sulfate, or hydroxyurea revealed antifungal effects against *C. neoformans*. Importantly, combination treatment with bortezomib and flucytosine in a murine inhalation model of cryptococcosis resulted in the improvement of neurological functions and reduced fungal replication and dissemination, leading to a delay in disease progression.

## RESULTS

### Bortezomib inhibits proteasome activity, proliferation, and capsule elaboration

**Fig 1:**
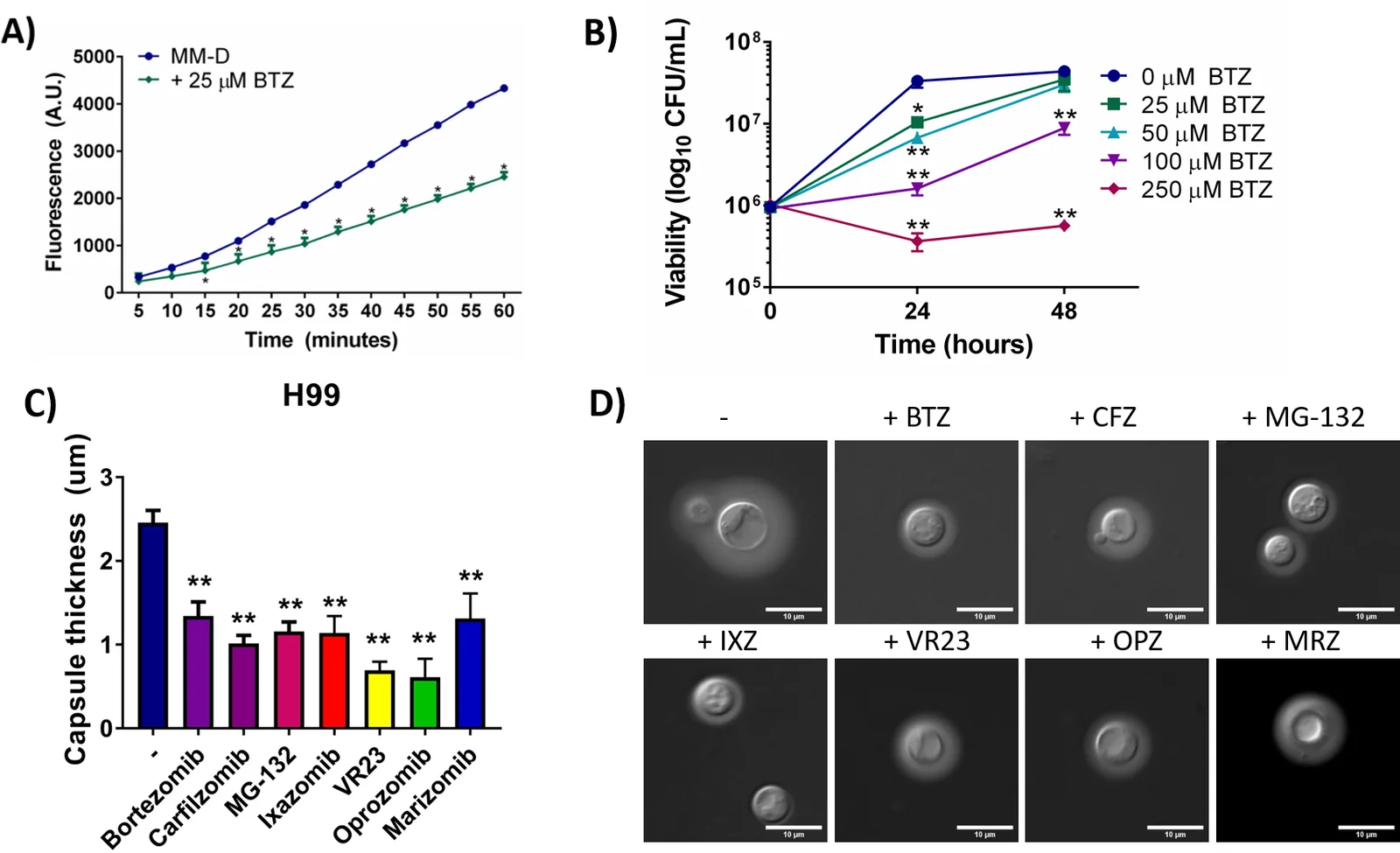
Proteasome inhibitors impair chymotrypsin-like peptidase activity, growth, and capsule formation in *Cryptococcus neoformans*. (**A**) Chymotrypsin-like peptidase activity of the proteasome was measured by the cleavage of SUC-LLVY-AMC peptide in the presence of bortezomib (BTZ). The averages for four biological replicates are shown. (**B**) Growth inhibition and impaired survival of H99 were observed in the presence of various concentration of bortezomib in minimal media at 37°C (average of three biological replicates, * *P ≤* 0.05, ** *P ≤* 0.01). (**C**) The impact of proteasome inhibitors on capsule formation was assessed for the WT strain (H99) grown in minimal medium for 48 h with or without 50 µM of the indicated inhibitors at 37°C. Assays were performed in triplicate, **P ≤ 0.001. (**D**) Representative images of reduced capsule size upon treatment with proteasome inhibitors (50 µM) during growth for 48 h in capsule-inducing medium. Capsule size was assessed by India ink staining. Abbreviations are as follows: bortezomib (BTZ), carfilzomib (CFZ), ixazomib (IXZ), oprozomib (OPZ), and marizomib (MRZ). Ordinary two-way ANOVA was used for statistical analysis of Chymotrypsin-like peptidase activity of the proteasome and growth inhibition, and one-way ANOVA was used for statistical analysis of capsule size.

In eukaryotic cells, the conserved 26S proteasome is a multi-subunit complex composed of a 20S CP and a 19S RP (23, 28). Amino acid sequence comparisons of 33 proteins that form the CP and the RP complexes revealed that the proteasome machinery is highly conserved between *C. neoformans* strain H99 and *Saccharomyces cerevisiae* strain S288c (Table S1). In particular, the predicted polypeptide sequence encoded by CNAG_05770 showed >73% identity with Pre2 encoding the β5 subunit targeted by the proteasome inhibitor bortezomib in yeast. We previously demonstrated that bortezomib impairs capsule formation and influences the growth of *C. neoformans* strains with altered expression of protein kinase A subunits (20). To extend our analysis, we initially assessed the ability of bortezomib to inhibit the catalytic activity of the proteasome in *C. neoformans* cells. For this experiment, the wild-type (WT) strain H99 was grown to stationary phase and exposed to bortezomib for 2 h prior to measuring chymotrypsin-like peptidase activity in lysates by cleavage of the fluorogenic substrate SUC-LLVY-AMC. The results revealed that proteasome activity was significantly reduced upon exposure to bortezomib compared to cells without drug treatment (Fig. 1A). This result suggested that the proteasome of *C. neoformans* possesses a chymotrypsin-like activity that can be inhibited by bortezomib.

Furthermore, we evaluated the impact of proteasome inhibition on proliferation and survival by growing cells in the presence of different concentrations of bortezomib (0–250 µM) at 37°C for 48 h. Colony-forming units (CFUs) were determined for the cultures at 0, 24, and 48 h, and the results indicated that bortezomib displayed a dose-dependent fungistatic effect on proliferation (Fig. 1B). We determined that the minimal inhibitory concentrations at 90% (MIC_90_) and 50% (MIC_50_) were 128 and 64 µg/mL, respectively. Taken together, the results suggest that proteasome machinery is required for cellular growth and viability. We also extended our evaluation of the impact of proteasome inhibition on capsule formation (20) by testing a suite of six different inhibitors. These inhibitors included the well-characterized reagent MG-132 and the inhibitors carfilzomib and ixazomib that are FDA-approved. As with bortezomib, each of these inhibitors caused a reduction in capsule size upon growth of cells in minimal medium (MM) for 48 h (Fig. 1C and D).

### Screens for mutants with altered bortezomib sensitivity

**Fig 2:**
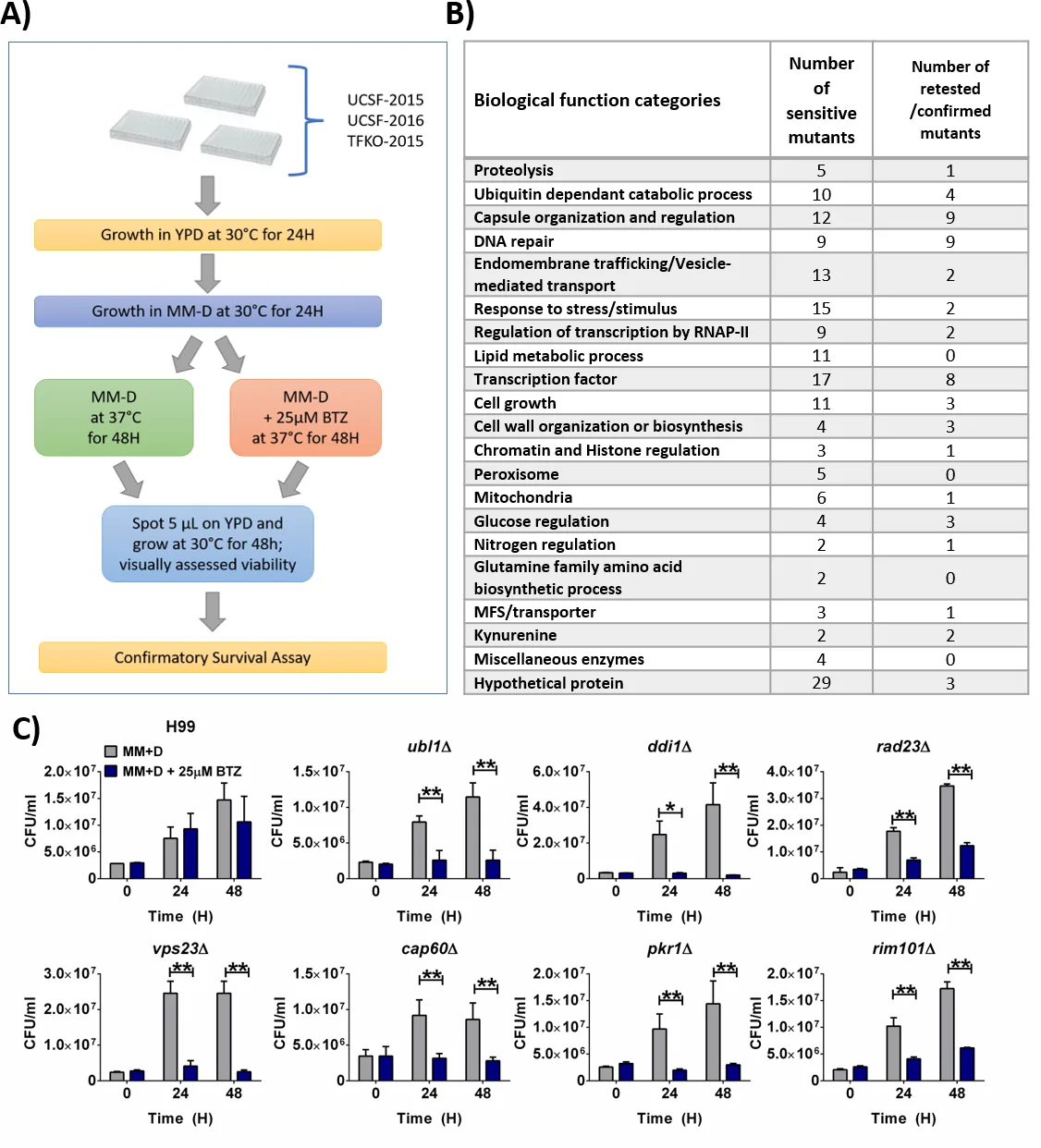
Chemical genetic screen of a collection of 4,221 deletion mutants. (**A**) Schematic of the screen of mutant libraries with the proteasome inhibitor Bortezomib (BTZ) at 37°C. (**B**) Summary of mutants with sensitivity to bortezomib classified by biological function. (**C**) Confirmation of bortezomib sensitivity by determining CFUs in minimal medium at 37°C at time 0, 24, and 48 h in the presence of 25 µM bortezomib compared to minimal medium without bortezomib for selective mutants. Strains with defects in proteolysis (*ubl1*Δ), ubiquitindependent catabolic processes (*vps23*Δ, *rad23*Δ, and *ddi1*Δ), capsule organization (*cap60*Δ), or regulation (*pkr1*Δ and *rim101*Δ) were tested. Multiple unpaired *t* test analyses were performed (*P < 0.05, **P < 0.01) for the confirmatory assays with the mutants.

To expand our understanding of the impact of proteasome inhibition on *C. neoformans*, we performed two chemical genetic screens of the available whole-genome deletion mutant collection. In the first screen, 4,221 deletion mutants were tested for survival after 48 h in the presence of 25 µM bortezomib in minimal media at 37°C. For this screen, we employed visual inspection of growth in a spot assay on solid medium after drug exposure, with subsequent assays of viability of selected mutants upon growth in liquid medium with bortezomib (Fig. 2A). We found 113 mutants that showed greater sensitivity than the WT strain (Table S2). These sensitive mutants were then classified and curated according to their biological and molecular functions according to the FungiDB database (https://fungidb.org) (Fig. 2B; Table S2). Although a number of genes could be classified in more than one category (Table S2), we observed several genes in the categories of proteolysis, ubiquitin-dependent catabolic processes, and capsule organization and regulation, thus supporting our previous observation of the involvement of the proteasome in capsule formation (20). As mentioned, the sensitivity to bortezomib of a selected set of mutants was validated by growth and viability assays (Fig. 2C), and we also confirmed the sensitivities of an additional 35 mutants with the same approach (Fig. S1). Interestingly, mutants in categories such as DNA repair, regulation of transcription by RNA polymerase II, lipid metabolic process, nitrogen regulation, and glutamine family amino acid biosynthetic process were confirmed. As discussed below, these categories showed an intriguing overlap with the categories identified in a screen for flucytosine sensitivity in *S. cerevisiae* (29).

We performed a second screen against bortezomib at 30°C with a total of 4,755 mutants and identified 153 and 261 mutants showing increased or decreased sensitivity, respectively (Table S3). The mutants that displayed increased sensitivity in both screens included strains with defects in ESCRT pathway components (e.g., Vps23), transcription factors (e.g., Rim101), DNA damage repair (e.g., Ercc-5), and capsule formation (e.g., Cap60). The latter result was particularly interesting given the observed impact of proteasome inhibition on capsule formation. The sensitivity of representative mutants lacking these proteins was confirmed in the subsequent growth assays along with the mutants validated from the first screen (Fig. 2C; Fig. S1).

### Selected tests of drug combinations with bortezomib for fungicidal activity

**Fig 3:**
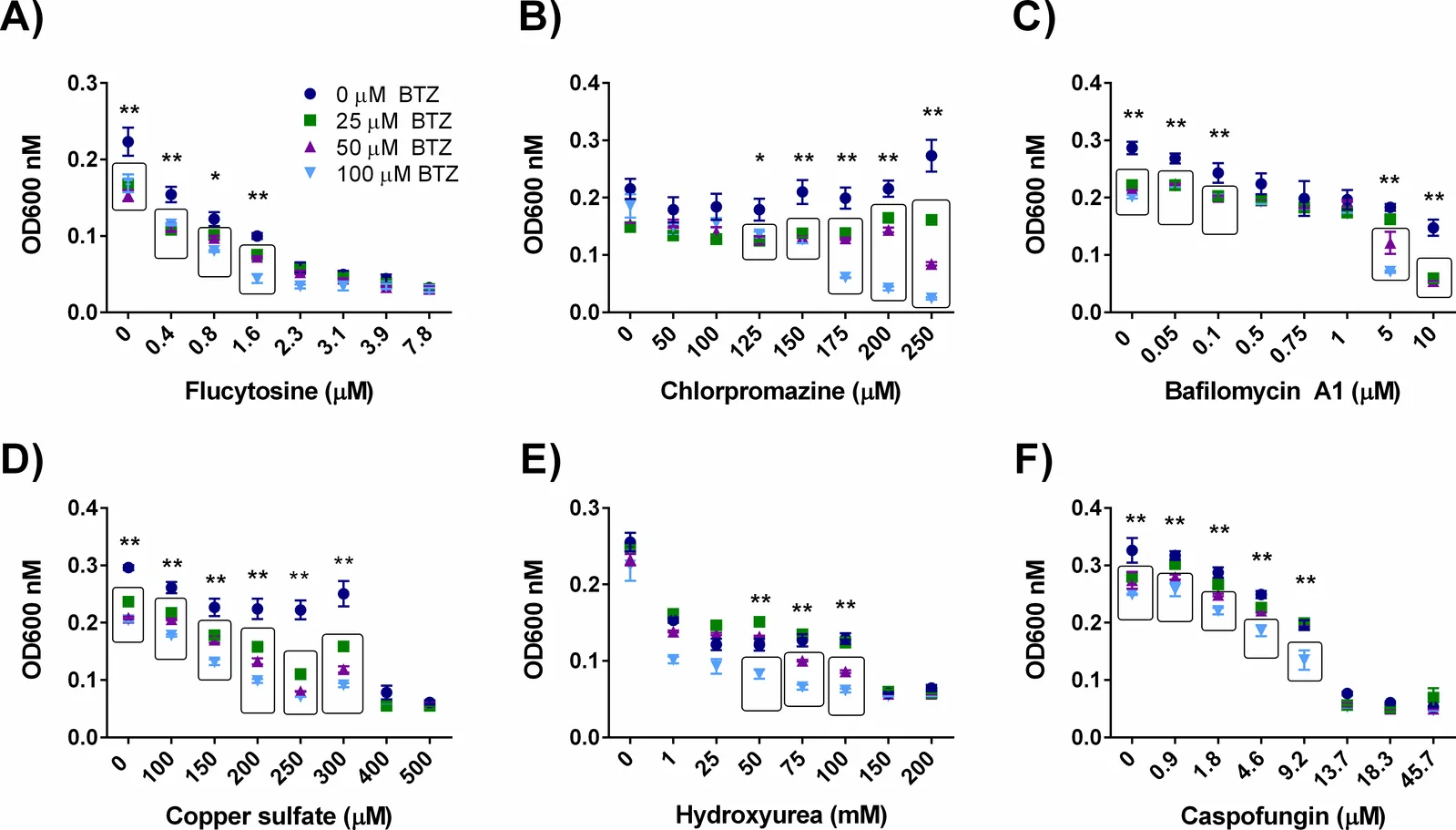
Inhibition of growth by combinations of bortezomib with other antifungal agents. Optical density was measured to assess growth inhibition when 25, 50, or 100 µM bortezomib (BTZ) was combined with the indicated concentrations of flucytosine (**A**), chlorpromazine (**B**), bafilomycin A1 (**C**), copper sulfate (**D**), hydroxyurea (**E**), and capsofungin (**F**). Ordinary two-way ANOVA was used for statistical analyses (*P < 0.05, **P < 0.01). Boxed results denote statistical significance compared to the untreated control (0 µM BTZ).

We next hypothesized that combinations of known fungistatic drugs could have an additive or synergistic effect, and potentially be fungicidal, when tested in combination with bortezomib. We selected compounds and drugs for these assays that target functions associated with sensitivity to bortezomib in our initial screen (Fig. 2). Measurements of growth by optical density showed a significant inhibition when 25, 50, or 100 µM concentrations of bortezomib were combined with different concentrations of flucytosine (inhibition of DNA and protein synthesis) (30), chlorpromazine (inhibition of endocytosis), bafilomycin A1 (inhibition of vacuolar-type H^+^ ATPase) (31), copper sulfate (impaired catalase and peroxidase activity) (32), hydroxyurea (DNA damage) (33), or caspofungin (inhibition of cell wall biosynthesis) (34). In general, combinations with bortezomib showed a greater reduction in growth than individual drugs or compounds (Fig. 3A through F). The fractional inhibitory concentration (FIC) indexes were calculated and found to be between 1 and 2 for all combinations, which suggested that the effects on growth were indifferent, but not antagonistic. The Bliss model was employed to look more closely at the inhibitory interactions between BTZ and the other agents. This analysis revealed additive interactions for all combinations except copper sulfate treatment, for which potential synergy was detected (Table 1).

**TABLE 1:** Bliss synergy scores of drugs with bortezomib^*a*^

Drug	Bliss synergy score	Interpretation
Flucytosine	8.51 ± 1.48	Additive
Chlorpromazine	4.21 ± 2.82	Additive
Hydroxyurea	5.88 ± 1.72	Additive
Copper sulfate	18.11 ± 1.54	Synergistic
Bafilomycin A1	3.35 ± 1.89	Additive
Caspofungin	0.91 ± 2.27	Additive

^*a*^ Synergy scores were determined by the method of Ianevski et al., (35).

**Fig 4:**
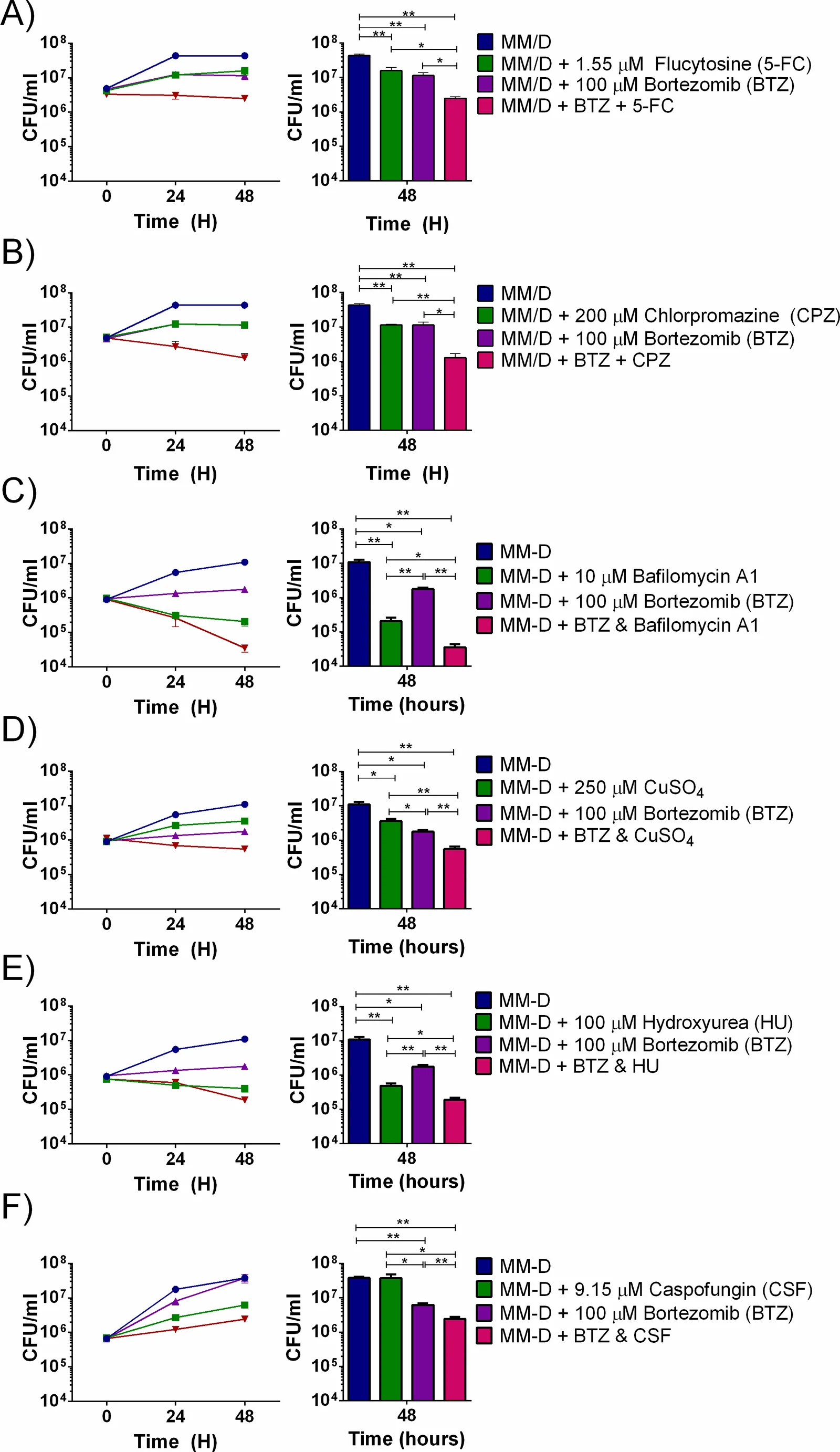
Evaluation of fungicidal activities for combinations of bortezomib with other antifungal agents. CFU counts were determined for cells in minimal medium at 37°C for 48 h in the presence of 100 µM bortezomib (BTZ) in combination with (A) 200 ng/mL flucytosine, (B) 200 µM chlorpromazine, (C) 10 µM bafilomycin A1, (D) 250 µM copper sulfate, (E) 100 µM hydroxyurea, or (F) 10 µg/mL caspofungin. Multiple unpaired *t* tests were used for statistical analyses (*P < 0.05, **P < 0.01).

Growth and viability were next assessed for the drug combinations by evaluating CFUs at 48 h (Fig. 4). As expected, each drug or compound except caspofungin reduced the growth of the WT strain alone compared to untreated controls, confirming their fungistatic effect on *C. neoformans*. However, a fungicidal effect was observed for all combinations, with CFUs significantly lower at 48 h when compared to the number of viable cells of the untreated control, and when compared to the CFUs of cells exposed to only one drug at 48 h. These results suggest a fungicidal effect when bortezomib is combined with flucytosine (Fig. 4A), chlorpromazine (Fig. 4B), bafilomycin A1 (Fig. 4C), copper sulfate (Fig. 4D), hydroxyurea (Fig. 4E), or caspofungin (Fig. 4F). Taken together, the screen for mutants with increased sensitivity to bortezomib reinforced the involvement of the proteasome in growth and survival, and identified biological functions with the potential to be druggable targets for combination treatment.

### The combination of bortezomib and flucytosine influences disease progression in mice

**Fig 5:**
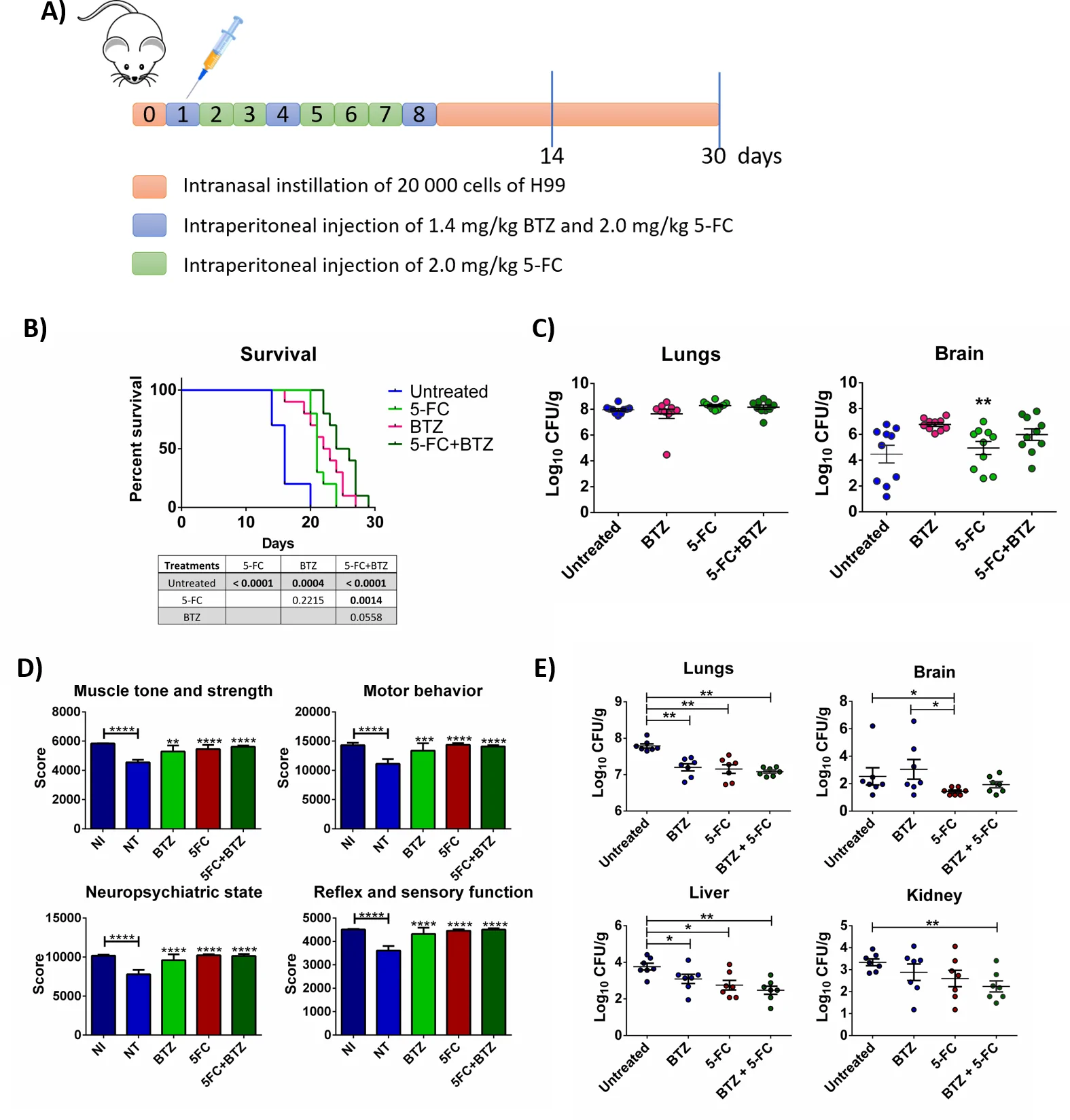
Evaluation of survival, fungal burden and neurological functions for infected mice treated with bortezomib, flucytosine, or both drugs. (**A**) Schematic of the treatment regimen of mice with bortezomib and flucytosine. A total of 10 mice were used for each group. (**B**) Survival of mice from each treatment group. Ordinary one-way ANOVA was used for statistical analyses and the values are presented in the table below the graph. (**C**) Fungal burden at time of death (lungs and brain) of infected mice treated with BTZ, 5-FC, or both drugs. (**D**) Improvement of neurological functions in infected mice treated with BTZ, 5-FC, or both drugs compared to infected and non-treated mice by behavioral assessment (SHIRPA) (37, 38). The data were analyzed by ANOVA (*P < 0.05, **P < 0.01, ***P = 0.0002, and ****P < 0.0001). (**E**) Reduced fungal load at 14 days post-infection in lungs, brain, liver, and kidney in infected mice treated with BTZ, 5-FC, or both. Unpaired Mann-Whitney *t* test was used for statistical analyses of fungal burden (*P < 0.05, **P < 0.01).

Flucytosine is commonly used in combination with amphotericin B for the treatment of cryptococcosis (36). The fungicidal effect of bortezomib and flucytosine observed in our *in vitro* assays prompted an evaluation of the efficacy of the drug combination in a murine model of cryptococcosis. Initially, we used five groups of mice for the experiment. One uninfected group was a control and four groups were inoculated intranasally with the WT strain. Of these, one group was not treated with drugs, and the other three groups were subjected to treatment with 5-FC or bortezomib alone, or the combination of the drugs at 24 h post-infection. Treatments involved daily doses of 100 mg/kg 5-flucytosine and/or three doses of 1.4 mg/kg bortezomib (at days 1, 4, and 8) administered intraperitoneally (Fig. 5A). The treatment regiments ended on day 8 post-inoculation and mice were left untreated for the rest of the experiment. The infected and untreated group succumbed to cryptococcosis between days 14 and 20 post-inoculation (Fig. 5B). Treatment with either flucytosine or bortezomib alone significantly prolonged survival when compared to the untreated group. The combination treatment with bortezomib and flucytosine also significantly extended survival when compared with the untreated mice or those treated with flucytosine alone (Fig. 5B). All groups eventually developed cryptococcosis with similar fungal loads in the lungs but with different loads in the brain at time of death (Fig. 5C). In particular, mice receiving bortezomib treatment alone displayed greater fungal burden in the brain. A more detailed behavioral assessment of the mice, including muscle tone and strength, motor behavior, neuropsychiatric state, and reflex and sensory function, indicated improved neurological function in mice treated with bortezomib, flucytosine, or both when compared to the infected and non-treated groups (Fig. 5D). No significant differences were observed among the groups for autonomous function (data not shown).

Given the fungal load differences in the brain at the time of death, we repeated the experiment with the same treatment regimen to assess the fungal load for each group at day 14 post-infection, when the untreated mice started to succumb to the infection. The results indicated a significant reduction in the fungal burden in the lungs, liver, and kidneys of mice treated with bortezomib and flucytosine (Fig. 5E). In contrast, the fungal loads in the brain (Fig. 5D) were lower when compared to the loads determined at the time of death (Fig. 5B), and no significant reductions were seen in untreated mice or mice treated with bortezomib alone (Fig. 5D). These results suggest that a distinct route of dissemination to the brain might escape the effect of bortezomib, or that this drug has other influences (e.g., on the immune response). Taken together, these results suggest that treatment with bortezomib in combination with flucytosine has a beneficial impact on mice physiology, and reduces initial fungal replication and dissemination leading to a delay in disease progression. A parallel experiment performed with a combination of BTZ and amphotericin B did not reveal improved survival for infected mice (Fig. S2).

## DISCUSSION

In this study, we characterized the antifungal activity of the FDA-approved proteasome inhibitor bortezomib against *C. neoformans* and identified mutants with altered sensitivity in genome-wide screens performed at 30°C or 37°C. We focused on validating a subset of sensitive mutants, including ones that appeared in both screens, in viability assays upon incubation with bortezomib. This analysis guided and supported the further analysis of the potential fungicidal activity of bortezomib in combination with flucytosine, chlorpromazine, bafilomycin A1, copper sulfate, hydroxyurea, or caspofungin. Importantly, administration of bortezomib in combination with flucytosine improved neurological function, reduced fungal burden in lungs, and prolonged survival for infected mice. The impact of bortezomib alone warrants further study because we found differences in the fungal burden in the brain compared with other treatments, yet improved neurological functions were observed.

Combination therapies are widely used for treatment of chronic infectious diseases such as AIDS/HIV, tuberculosis, and malaria (39 – 41). This strategy has been applied on a limited basis for treatment of cryptococcosis with antifungal drugs (42). For example, the clinical guidelines for treatment of cryptococcal meningitis consist of regimens of amphotericin B, flucytosine, and fluconazole, alone or in combination (42). Considered as the gold standard, patients receiving combination therapy of amphotericin B and flucytosine need to be closely monitored for the renal toxicity associated with amphotericin B (43). Furthermore, accessibility to flucytosine is limited in underserved communities, and the use of amphotericin B and fluconazole alone or in combination is often an inferior alternative (2). Therefore, failure of clearance of cryptococcal cells and nephrotoxicity often jeopardize treatment and result in poor outcomes for patients (43 – 45). Additionally, resistance to flucytosine rapidly develops during monotherapy, thus emphasizing the need to expand the current arsenal of antifungals including strategies for combination therapy.

Successful new approaches for treatment of fungal diseases include counteracting known virulence factors, targeting known resistance mechanisms for current antifungal drugs, and systematic screening for effective combinations of novel or repurposed drugs (45, 46). In this regard, bortezomib may act in part to counteract the contribution of the capsule to virulence, and we demonstrated in this study that several proteasome inhibitors impact capsule formation. In this context, there are examples of successful combinations including pairing the statin drug atorvastatin with fluconazole to alter capsule properties of *Cryptococcus gattii* (46). In an experimental model of infection, mice treated with atorvastatin and fluconazole showed improved clinical conditions, reduced fungal burden in the lungs and brain, and increased survival (46). Likewise, the antifungal activity of the flavonoid compound of turmeric (curcumin) paired with fluconazole enhanced neurologic functions, reduced pulmonary damage and fungal burden in the brain, and increased survival in mice infected with *C. gattii* (47). Moreover, combination therapy with 5-flucytosine and an IgG1 monoclonal antibody to *C. neoformans* capsule resulted in a reduction in fungal burden in the lungs (48, 49). Passive immunotherapy with a monoclonal antibody directed against the capsular polysaccharide of *C. neoformans* was also a promising therapeutic avenue that warranted a phase I clinical trial; however, this approach was not further developed due to insufficient resources (48, 49). In terms of targeting resistance, the natural compound beauvericin blocks the multidrug efflux pump Pdr5 and inhibits the global regulator TORC1 kinase (50). The lifespan of mice infected with *Candida albicans* was extended upon administration of beauvericin in combination with fluconazole (51). Furthermore, the FK506 analog 9-deoxo-31-O-demethyl-FK506 had strong antifungal activity by targeting the calcineurin pathway in *C. neoformans* (51). When combined with fluconazole, 9-deoxo-31-O-demethyl-FK506 exhibited synergistic activity and significantly extended the survival of mice infected with *C. neoformans* (51).

In the recent years, a successful method to identify novel synergistic drug combinations with current antifungal drugs was through large-scale systematic screens of compound libraries. Several chemical biology screens identified inhibitory drug combinations against *S. cerevisiae*, *S. pombe*, *C. albicans*, *Aspergillus fumigatus,* and *C. neoformans* (52 – 59). For example, Robbins et al., generated an Antifungal Combinations Matrix by evaluating the growth of *S. cerevisiae*, *S. pombe*, *C. albicans*, and *C. neoformans* in the presence of sub-lethal concentrations of known antifungals (i.e., fluconazole, caspofungin, amphotericin B, terbinafine, benomyl, and cyprodinil) in combination with 3,600 compounds (56). This generated a rich reservoir of chemical-chemical interactions with therapeutic potential against several human fungal pathogens. Systematic screens with off-patent drugs identified compounds that potentiate fluconazole action in *Candida*, *Cryptococcus*, and *Saccharomyces* strains (54). These screens revealed two classes of synergistic compounds that either perturbed membrane permeability or inhibited sphingolipid biosynthesis (54). Sphingolipid antagonists were further established as a new class of antifungal agents (58). Large-scale systematic screens were also conducted on small molecule libraries on deletion mutant collections of *C. neoformans* (59). Chemical-genetic response of *C. neoformans* predicted novel antifungal synergies with fluconazole, novel pathogenicity genes, and inferred modes of action for some compounds (59). These studies highlight the utility of high-throughput screens to identify novel combinatory therapy to treat fungal infections. We also note that inhibition of proteasome activity has emerged as a powerful strategy for anticancer therapy, but also shows promise for treating tuberculosis, malaria, leishmaniasis, Chagas disease, sleeping sickness, and schistosomiasis (60 – 67). Structure-function studies of the proteasome of *Mycobacterium tuberculosis* and *Plasmodium falciparum* resulted in greater selective inhibition of bacterial and parasitic proteasome relative to the human proteasome (61). Furthermore, cell-based screening of diverse chemical scaffolds yielded compounds with high selectivity to parasitic proteasome and low host toxicity (62 – 64). Taken together, these studies indicate that the development of selective proteasome inhibitors as anti-infective agents is a novel therapeutic avenue for diseases with limited treatment options.

In summary, our study revealed the potential of proteasome inhibition to impair fungal proliferation in vitro and to influence the outcome of experimental cryptococcosis*.* Further investigations are needed to understand the precise impact of proteasome inhibition on the different biological functions uncovered in this study. Understanding these mechanisms will potentially allow the application of proteasome inhibitors as both anti-virulence and anti-proliferation agents against cryptococcosis. This work also highlights the utility of chemical genetic screens to identify new therapeutic avenues, a strategy that aligns well with the global effort to expand the current arsenal of antifungal drugs.

## MATERIALS AND METHODS

### Strains and media

The serotype A strains H99 and KN99 of *C. neoformans* var. *grubii* were used as WT strains and routinely maintained on YPD medium (1% yeast extract, 2% peptone, 2% dextrose, and 2% agar). Chemical screens and proteasome assays were performed with cells grown in MM (15.0 mM glucose, 6.5 mM (NH_4_)_2_SO_4_, 10.0 mM MgSO_4_, 29.4 mM K_2_HPO_4_, and 3.0 µM thiamine, pH 5.4) at 30°C or 37°C (68). Low iron, capsule-inducing medium (CIM) was prepared using Chelex-100-treated NANOpure water (0.5% dextrose, 0.4 g/L dipotassium monohydrogen phosphate, 5 g/L asparagine, 0.25 g/L calcium chloride dehydrate, 0.4 mg/L thiamine, 0.005 mg/L cupric sulfate pentahydrate, 2 mg/L zinc sulfate heptahydrate, 0.01 mg/L manganese chloride tetrahydrate, 80 mg/L magnesium sulfate heptahydrate, 0.46 mg/L sodium molybdate, and 0.057 mg/L boric acid) (68).

### Proteasome activity assay

Cells were grown overnight at 37°C in MM, washed two times in phosphate-buffered saline (PBS), counted, resuspended in MM +25 µM bortezomib and placed at 37°C for 2 h. After treatment, cells were washed two times in PBS and resuspended in 300 µL of filtered-sterilized lysis buffer [50 mM HEPES (pH 7.8), 10 mM NaCl, 1.5 mM MgCl2, 1 mM EDTA, 1 mM EGTA, 250 mM sucrose, 5 mM DTT, 1 mM 4-(2-aminoethyl) benzenesulfonyl fluoride hydrochloride, and 50 µM E-64]. Ccells were disrupted in a Retsch MM301 mixer mill with glass beads for 5 min, and then vortexed six times in intervals of 1 min. Cell lysates were then centrifuged for 15 min at 13,000 rpm at 4°C and the total protein content of supernatants was determined by Bradford assay. Protein concentrations were adjusted to 200 µg/mL and 25 µL of each sample was added to 100 µL buffer assay (lysis buffer + 2 mM ATP) with 100 µM substrate (SUC-LLVY-AMC) for 60 min at 37°C; fluorescence was read at 360–460 nm on a Tecan Infinite M200 plate reader.

### Growth assays

To assess growth in liquid media, cells were pre-grown in liquid YPD at 30°C for 20 h, washed three times in PBS, and sub-cultured in MM for 20 h at 30°C. Cells were then washed three times in PBS and 1 × 10^6^ cells/mL were inoculated in triplicate cultures of MM with different concentrations (25–250 µM) of bortezomib, followed by incubation at 37°C for 48 h. CFUs were counted from culture samples at 0, 24, and 48 h.

### Capsule formation

Cells were pre-grown in YPD for 24 h at 30°C, washed three times in PBS, and 3 × 10^6^ cells/mL were inoculated into low-iron, CIM for 48 h at 37°C. Cells were then stained with India ink and capsule formation was examined by differential interference contrast microscopy at 1,000× magnification with an Axio imager M.2 microscope (Zeiss). Capsule size measurements were performed using ImageJ. The impact of proteasome inhibitors on capsule formation was assessed using 50 µM concentrations of bortezomib, carfilzomib, MG-132, ixazomib, VR23, oprozomib, and marizomib. The inhibitors were obtained from Cell Signaling Technology, USA or Selleckchem, USA.

### Screens for bortezomib sensitive mutants

Two screens were performed to identify mutants with altered sensitivity to bortezomib using the *C. neoformans* mutant collections from the laboratories of Dr. H. Madhani and Dr. Y.-S. Bahn, available from the Fungal Genetics Stock Center (69 – 71; https://www.fgsc.net). The first screen was performed at 37°C and employed 4,221 mutants. The mutants were grown overnight in YPD at 30°C in 96-well plates, and 5 µL from each of culture was inoculated into 195 µL of MM and placed at 30°C overnight. From each culture, 5 µL was inoculated into 95 µL of MM ±25 µM bortezomib and placed at 37°C for 48 h. Optical density at 600 nm was taken after 48 h with a Tecan plate reader and viability was assessed by spotting 5 µL onto YPD plates with incubation at 30°C for 48 h. Sensitive mutants showed no growth or markedly reduced growth on solid medium compared to the control WT strain. A subset of mutants with growth defects in the presence of bortezomib was subsequently confirmed by viability assays in liquid medium using CFU counts.

The second screen was performed by growing the mutants from the libraries overnight in YPD at 30°C in 96-well plates. From the YPD cultures, 5 µL was inoculated into 195 µL of MM with or without 25 µM bortezomib, and placed in a shaker at 30°C for 72 h. The OD_600_ was measured at 0 h, and after 72 h with a Tecan plate reader. Deletion mutants showing differential susceptibility to bortezomib were identified by assessing the growth differences at 72 h between the medium with and without drug. The OD_600_ values in these two media conditions were normalized with their respective blank media controls (OD_600_ at 0 h), and the relative growth difference (log2 ratio) for each strain was calculated. The mean log2 ratio of each plate was determined to evaluate the overall growth behavior of the strains in each individual plate. Strains with altered bortezomib susceptibility were identified through a cutoff of ±1.5 times the standard deviation (SD) from the mean log2 ratio of each 96-well plate. The strains with log2 ratios ≥mean log2 ratio +1.5 SD or ≤mean log2 ratio −1.5SD were selected as bortezomib-resistant and sensitive strains, respectively.

### Drug combination assays

To assess the effects of drug combinations on the growth of the WT strain H99, cells were pre-grown in liquid YPD at 30°C for 24 h, washed three times in PBS and inoculated at 1% in MM for an additional 24 h at 30°C. Cells were then washed three times in PBS, counted and 1 × 10^6^ cells/mL were inoculated in triplicate in 96-well plates into MM with different concentrations (0, 25, 50, and 100 µM) of bortezomib and gradient concentrations of flucytosine (0–1,000 ng/mL), chlorpromazine (0–250 µM), bafilomycin A1 (0–10 µM), copper sulfate (0–500 µM), or hydroxyurea (0–200 mM). The drugs and chemicals were obtained from MilliporeSigma, Canada. Plates were incubated at 37°C for 48 h and optical density was measured at 600 nm on a Tecan Infinite M200 plate reader. To examine fungicidal effects, survival in liquid cultures at 37°C was assessed. Briefly, cells were pre-grown in liquid YPD at 30°C for 20 h, washed three times in PBS and inoculated at 1 × 10^6^ cells/mL in triplicate cultures of MM with and without 100 µM of bortezomib and additional drug (i.e., 200 ng/mL flucytosine, 200 µM chlorpromazine, 10 µM bafilomycin A1, 250 µM copper sulfate, or 100 µM hydroxyurea). Liquid cultures were incubated at 37°C for 48 h and CFUs were assessed at 0, 24, and 48 h by plating serial dilutions on solid YPD medium. ANOVA tests were performed on each data set and compared to the control without bortezomib.

The potential synergy between drug treatments was assessed using the Bliss model (35) and online tools (https://synergyfinder.fimm.fi). Dose–response matrices were calculated for each drug combination to generate Bliss synergy scores where a score <−10 indicates a likely antagonistic interaction, a score from −10 to 10 indicates a likely additive interaction, and a score >10 indicates potential synergy.

### Virulence assays in mice

Survival assays were performed using female BALB/c mice (4–6 weeks old) from Charles River Laboratories (Ontario, Canada) infected with *C. neoformans* strain H99. Mice were anesthetized intraperitoneally with ketamine (80 mg/kg) and xylazine (5.5 mg/kg) and suspended on a silk thread by the superior incisors. Fungal cells were grown in 5 mL of YPD at 30°C and washed two times with PBS. A suspension of 2 × 10^4^ cells in 50 µL was slowly inoculated into the nares of the mice. The health status of the mice was monitored daily post-inoculation and mice reaching the humane endpoint were euthanized by CO_2_ anoxia. Different treatment regimens were performed to assess the impact of drug treatment on disease progression. Mono and combination treatments began at 24 h post-infection and continued over a period of 8 days. Daily doses of 5-flucytosine (100 mg/kg) and three doses of bortezomib (at days 1, 4, and 8) (1.4 mg/kg) were administered intraperitoneally.

During the survival analysis, morbidity was monitored by determining mice behavioral profiles using the SHIRPA protocol (Smith Kline/Harwell/Imperial College/Royal Hospital/Phenotype Assessment-SHIRPA) (37, 38). The protocol is based on tests that provide quantitative data through a score on functional performance. This performance is compared between animals and over time between groups. The parameters analyzed were grouped into five distinct categories according to Table 2: neuropsychiatric status, motor behavior, muscle tone and strength, reflex and sensory function, and autonomous function (37, 38).

**TABLE 2:** Functional categories of SHIRPA protocol according to the analyzed parameters

Functional categories	Parameters (38)
Muscle tone and strength	Grip strength, body tone, limb tone, abdominal tone
Motor behavior	Body position, tremor, locomotor activity, pelvic elevation, gait, tail elevation, trunk curl, limb grasping, wire maneuver, negative geotaxis
Neuropsychiatric state	Spontaneous activity, transfer arousal, touch escape, positional passivity, biting, fear, irritability, aggression, vocals
Autonomous function	Respiration rate, defecation, urination, palpebral closure, piloerection, skin color, heart rate, lacrimation, salivation
Reflex and sensory function	Startle response, visual placing, pinna reflex, corneal reflex, toe pinch, righting reflex

After the survival and behavior experiments, additional groups of mice were infected to determine the fungal burden in organs (lungs, brain, liver, and kidney) at 14 days post-infection. Organs were removed aseptically, weighed, and homogenized using a Retsch MM301 mixer mill. The samples were serial diluted in PBS, plated on YPD medium containing chloramphenicol (30 µg/mL) and incubated at 30°C for 2 days. CFUs were then counted. Statistical analyses of survival differences and fungal burden in mice were performed with the log rank test and a two-tailed unpaired Mann-Whitney test, respectively (GraphPad Prism 8 for Windows, GraphPad Software, San Diego, CA, USA).

## SUPPLEMENTAL MATERIAL

Supplemental Tables S1 to S3, Fig. S1 and S2 spectrum.01904-23-s0001.pdf Supplemental tables and figures.

ASM does not own the copyrights to Supplemental Material that may be linked to, or accessed through, an article. The authors have granted ASM a non-exclusive, world-wide license to publish the Supplemental Material files. Please contact the corresponding author directly for reuse.
