# Supplementary material for: Proteasome inhibition as a therapeutic target for the fungal pathogen Cryptococcus neoformans
Source: Microbiol Spectr. 2023 Sep 26;11(5):e01904-23. doi: 10.1128/spectrum.01904-23 (PMC10580939; doi:10.1128/spectrum.01904-23)
Supplement: Supplemental Tables S1 to S3, Fig. S1 and S2 — Supplemental tables and figures. [file spectrum.01904-23-s0001.pdf]

**Supplemental Table 1:** Summary of protein sequence comparisons of the conserved 26S proteasome machinery between *C. neoformans* H99 and *Saccharomyces cerevisiae* S288c. Thirty-three proteins that forms the CP and the RP complexes in *S. cerevisiae* S288c were found in *C. neoformans* using NCIB Blastp.

| Complex | Protein name | Locus tag *C. neoformans* H99 | Systematic name *S. cerevisiae* S288c | E value | Per. Ident | Description |
|---|---|---|---|---|---|---|
| **20S core particle (CP)** | SCL1 | CNAG_04001 | YGL011C | 9.00E-82 | 45.98% | proteasome core particle subunit alpha 1 |
| **Alpha subunits** | PRE8 | CNAG_03941 | YML092C | 7.00E-104 | 63.11% | proteasome core particle subunit alpha 2 |
| | PRE9 | CNAG_05792 | YGR135W | 3.00E-96 | 50.57% | proteasome core particle subunit alpha 3 |
| | PRE6 | CNAG_01035 | YOL038W | 9.00E-117 | 63.86% | proteasome core particle subunit alpha 4 |
| | PUP2 | CNAG_05475 | YGR253C | 2.00E-116 | 66.67% | proteasome core particle subunit alpha 5 |
| | PRE5 | CNAG_04071 | YMR314W | 2.00E-82 | 55.05% | proteasome core particle subunit alpha 6 |
| | PRE10 | CNAG_05269 | YOR362C | 2.00E-88 | 53.16% | proteasome core particle subunit alpha 7 |
| **Beta subunits** | PRE3 | CNAG_03816 | YJL001W | 7.00E-97 | 63.98% | proteasome core particle subunit beta 1 |
| | PUP1 | CNAG_02725 | YOR157C | 2.00E-101 | 56.60% | proteasome core particle subunit beta 2 |
| | PUP3 | CNAG_00385 | YER094C | 3.00E-87 | 58.54% | proteasome core particle subunit beta 3 |
| | PRE1 | CNAG_02886 | YER012W | 5.00E-62 | 46.70% | proteasome core particle subunit beta 4 |
| | PRE2 | CNAG_05770 | YPR103W | 5.00E-123 | 73.28% | proteasome core particle subunit beta 5 |
| | PRE7 | CNAG_05865 | YBL041W | 4.00E-88 | 56.96% | proteasome core particle subunit beta 6 |
| | PRE4 | CNAG_01957 | YFR050C | 3.00E-74 | 44.44% | proteasome core particle subunit beta 7 |
| **19S regulatory particle (RP)** | | | | | | |
| **Lid** | RPN3 | CNAG_00062 | YER021W | 4.00E-77 | 29.88% | proteasome regulatory particle lid subunit RPN3 |
| | RPN5 | CNAG_01083 | YDL147W | 2.00E-92 | 36.22% | proteasome regulatory particle lid subunit RPN5 |
| | RPN6 | CNAG_06361 | YDL097C | 4.00E-98 | 41.49% | proteasome regulatory particle lid subunit RPN6 |
| | RPN7 | CNAG_06899 | YPR108W | 2.00E-79 | 39.13% | proteasome regulatory particle lid subunit RPN7 |
| | RPN8 | CNAG_00046 | YOR261C | 1.00E-92 | 45.26% | proteasome regulatory particle lid subunit RPN8 |
| | RPN9 | CNAG_04014 | YDR427W | 2.00E-78 | 34.83% | proteasome regulatory particle lid subunit RPN9 |
| | RPN11 | CNAG_07028 | YFR004W | 3.00E-148 | 65.69% | proteasome regulatory particle lid subunit RPN11 |
| | RPN12 | CNAG_03721 | YFR052W | 5.00E-19 | 31.34% | proteasome regulatory particle lid subunit RPN12 |
| | RPN13 | CNAG_02400 | YLR421C | 5.00E-05 | 31.31% | proteasome regulatory particle lid subunit RPN13 |
| | SEM1 | CNAG_06402 | YDR363W-A | 0.02 | 44.07% | proteasome regulatory particle lid subunit SEM1 |
| **Base** | RPN1 | CNAG_07863 | YHR027C | 0 | 42.07% | proteasome regulatory particle base subunit RPN1 |
| | RPN2 | CNAG_06175 | YIL075C | 0 | 38.95% | proteasome regulatory particle base subunit RPN2 |
| | RPN10 | CNAG_00482 | YHR200W | 4.00E-66 | 46.44% | proteasome regulatory particle base subunit RPN10 |
| | RPT1 | CNAG_07719 | YKL145W | 0 | 70.88% | proteasome regulatory particle base subunit RPT1 |
| | RPT2 | CNAG_02239 | YDL007W | 0 | 72.27% | proteasome regulatory particle base subunit RPT2 |
| | RPT3 | CNAG_03904 | YDR394W | 0 | 71.23% | proteasome regulatory particle base subunit RPT3 |
| | RPT4 | CNAG_04906 | YOR259C | 0 | 70.38% | proteasome regulatory particle base subunit RPT4 |
| | RPT5 | CNAG_06153 | YOR117W | 0 | 66.83% | proteasome regulatory particle base subunit RPT5 |
| | RPT6 | CNAG_04666 | YGL048C | 0 | 76.28% | proteasome regulatory particle base subunit RPT6 |

**Supplemental Table 2:** Classifications by biological function for mutants sensitive to bortezomib from the first screen.

| Biological Function | Protein name | Description | Confirmation in liquid medium |
|---|---|---|---|
| **Proteolysis** | | | |
| CNAG_00757 | Doa4 | Ubiquitin carboxyl-terminal hydrolase 8 | |
| CNAG_03872 | Ubp7 | Putative ubiquitin carboxyl-terminal hydrolase | |
| CNAG_05650 | Ubp5 | Ubiquitin carboxyl-terminal hydrolase 7 | |
| CNAG_05708 | Ubp14 | Ubiquitin carboxyl-terminal hydrolase 5/13 | |
| CNAG_04159 | Ubl1 | E3 ubiquitin-protein ligase | Confirmed |
| | | | |
| **Ubiquitin-dependent catabolic process** | | | |
| CNAG_01720 | Vps23 | ESCRT-I subunit protein VPS23 | Confirmed |
| CNAG_00248 | Vps36 | ESCRT-II subunit protein | |
| CNAG_04863 | Vps25 | ESCRT-II subunit protein | |
| CNAG_05410 | Vid24 | Glucose-induced degradation complex subunit | |
| CNAG_04809 | Cop9 | Signalosome catalytic subunit RRI1 | |
| CNAG_03039 | Ddi1 | DNA damage-inducible v-SNARE binding protein | Confirmed |
| CNAG_00772 | Rad23 | Putative UV excision repair protein | Confirmed |
| CNAG_00252 | | Elongin C homolog | Confirmed |
| CNAG_00171 | Pex2p | RING-finger peroxin and E3 ubiquitin ligase | |
| CNAG_04937 | Pex12 | C3HC4-type RING-finger peroxin and E3 ubiquitin ligase | |
| | | | |
| **DNA repair** | | | |
| CNAG_00328 | Ercc-5 | DNA excision repair protein | Confirmed |
| CNAG_00550 | Msh5 | DNA mismatch repair protein | Confirmed |
| CNAG_00772 | Rad23 | Putative UV excision repair protein | Confirmed |
| CNAG_00991 | Flap1 | Flap endonuclease 1 | Confirmed |
| CNAG_02490 | Mre11 | DNA repair protein | Confirmed |
| CNAG_06384 | Rad50 | DNA repair protein | Confirmed |
| CNAG_06793 | Exo1 | Exonuclease 1 | Confirmed |
| CNAG_06143 | AlkB | Alkylated DNA repair protein | Confirmed |
| CNAG_05116 | | DNA polymerase mu subunit | Confirmed |

| | | | |
|---|---|---|---|
| **Chromatin and Histone regulation** | | | |
| CNAG_04159 | Ubl1 | E3 ubiquitin-protein ligase | Confirmed |
| CNAG_01340 | Yta7 | ATPase | |
| CNAG_04048 | Arp4 | 53 kda brg1-associated factor b/Arp4p | |
| | | | |
| **Regulation of transcription by RNAP-II** | | | |
| CNAG_06648 | Rft1 | mRNA polymerase-associated protein | |
| CNAG_00252 | | Transcription elongation factor B, polypeptide 1 | Confirmed |
| CNAG_00761 | | Coiled-coil domain-containing protein 12 | |
| CNAG_04118 | Ctk1 | Catalytic (alpha) subunit of C-terminal domain kinase I (CTDK-1) | |
| CNAG_01622 | Ctk2 | Beta subunit of C-terminal domain kinase I (CTDK-I) | |
| CNAG_04545 | Ctk3 | Gamma subunit of the C-terminal domain kinase I (CTDK-I) | |
| CNAG_07311 | Bre2 | Set1/Ash2 histone methyltransferase subunit | |
| CNAG_01243 | Set101 | Histone-lysine N-methyltransferase | Confirmed |
| CNAG_01377 | Ubp13 | PAB-dependent poly(A)-specific ribonuclease subunit PAN2 | |
| | | | |
| **Endomembrane trafficking / Vesicle-mediated transport** | | | |
| CNAG_01720 | Vps23 | ESCRT-I subunit protein VPS23 | Confirmed |
| CNAG_00248 | Vps36 | ESCRT-II subunit protein | |
| CNAG_04863 | Vps25 | ESCRT-II subunit protein | |
| CNAG_01583 | Snf7 | ESCRT-III subunit protein | |
| CNAG_00361 | Rvs167 | Amphiphysin | |
| CNAG_02173 | Trs33 | Core component of TRAPP complexes I, II and IV | |
| CNAG_06129 | Tvp23 | Tlg2-Vesicle Protein | |
| CNAG_04678 | Ypk1 | Serine/threonine protein kinase | |
| CNAG_02676 | Vam7 | Vacuolar SNARE protein | |
| CNAG_05162 | Vam9 | Vacuolar H+ ATPase subunit e of the V-ATPase V0 subcomplex | |
| CNAG_03039 | Ddi1 | DNA damage-inducible v-SNARE binding protein | Confirmed |
| CNAG_00517 | Shr3 | Endoplasmic reticulum packaging chaperone | |

| | | | |
|---|---|---|---|
| CNAG_06622 | Moh1 | Protein of unknown function; possibly linked with vacuolar transport | |
| | | | |
| **Lipid metabolic process** | | | |
| CNAG_06594 | Kes1 | Oxysterol-binding protein | |
| CNAG_07527 | Alg12 | Dolichyl-P-Man:Man(7)GlcNAc(2)-PP-dolichol alpha-1,6-mannosyltransferase | |
| CNAG_00594 | Yeh2 | Sterol esterase | |
| CNAG_06080 | Sac1 | Phosphatidylinositol-3-phosphatase | |
| CNAG_04678 | Ypk1 | Serine/threonine protein kinase | |
| CNAG_03080 | Elo1 | Fatty acid elongase | |
| CNAG_00490 | Pot1 | Acetyl-CoA C-acyltransferase | |
| CNAG_03139 | Cho2 | Phosphatidylethanolamine N-methyltransferase | |
| CNAG_05815 | Skn1 | Beta-glucan synthesis-associated protein | |
| CNAG_07639 | | Triacylglycerol lipase | |
| CNAG_00861 | | 3-hydroxyacyl-CoA dehydrogenase | |
| | | | |
| **Peroxisome** | | | |
| CNAG_00516 | Pex7 | Peroxisomal signal receptor for peroxisomal matrix proteins | |
| CNAG_06024 | Pex14 | Central component of the peroxisomal importomer complex | |
| CNAG_00171 | Pex2 | RING-finger peroxin and E3 ubiquitin ligase | |
| CNAG_04937 | Pex12 | C3HC4-type RING-finger peroxin and E3 ubiquitin ligase | |
| CNAG_05721 | Mfe2 | Multifunctional beta-oxidation protein | |
| | | | |
| **Mitochondria** | | | |
| CNAG_02325 | | Mitochondrial protein | |
| CNAG_00576 | Imo32 | Mitochondrial protein | |
| CNAG_02891 | Rdl1 | Thiosulfate sulfurtransferase | |
| CNAG_02270 | Met2 | Homoserine O-acetyltransferase | Confirmed |
| CNAG_01238 | ArgJ | Arginine biosynthesis ArgJ, mitochondrial | |
| CNAG_03452 | Afg1 | Chaperone for cytochrome c oxidase subunits | |
| | | | |
| **Response to stress/stimulus** | | | |
| CNAG_00293 | Ras1 | G-protein signaling GTPase | |

| | | | |
|---|---|---|---|
| CNAG_04981 | Cat1 | Catalase 1 | |
| CNAG_00575 | Cat3 | Catalase 3 | |
| CNAG_00888 | Cnb1 | Calcineurin b, regulatory subunit | |
| CNAG_01602 | | IQ domain-containing calmodulin-binding protein | |
| CNAG_00156 | Crz1 | Zinc finger transcription factor | Confirmed |
| CNAG_04647 | Gsh2 | Glutathione synthetase | |
| CNAG_06873 | Oxp1 | 5-oxoprolinase | |
| CNAG_01588 | Pmp3 | Plasma membrane proteolipid 3 | |
| CNAG_01452 | Mat3 | Pheromone repeat protein | |
| CNAG_07856 | Bwc3 | Arylsulfotransferase (ASST) domain | |
| CNAG_06224 | Mln2 | Nuclear mouvement protein nudC | |
| CNAG_03670 | Ire1 | Serine-threonine kinase and endoribonuclease | Confirmed |
| CNAG_01431 | Hob1 | Homeobox domain | |
| CNAG_04263 | Bzp2 | Hypothetical protein; bZIP transcription factor | |
| | | | |
| **Capsule organization and regulation** | | | |
| CNAG_00600 | Cap60 | Capsule-associated protein | Confirmed |
| CNAG_00721 | Cap59 | Alpha-1,3-mannosyltransferase | Confirmed |
| CNAG_01283 | Cap5 | Beta-1,2-xylosyltransferase | |
| CNAG_06016 | Cap6 | Alpha-1,3-mannosyltransferase | |
| CNAG_07937 | Cas1 | O-acetyltransferase | Confirmed |
| CNAG_07554 | Cap10 | Capsular associated protein | Confirmed |
| CNAG_00570 | Pkr1 | cAMP-dependent protein kinase regulator | Confirmed |
| CNAG_05222 | Nrg1 | Transcriptional regulator | Confirmed |
| CNAG_05431 | Rim101 | pH-response transcription factor | Confirmed |
| CNAG_03843 | Ark1 | NAK protein kinase | Confirmed |
| CNAG_01720 | Vps23 | ESCRT-I subunit protein VPS23 | Confirmed |
| CNAG_01583 | Snf7 | ESCRT-III subunit protein | |
| | | | |
| **Cell wall organization or biogenesis** | | | |
| CNAG_00156 | Crz1 | Zinc finger transcription factor | Confirmed |
| CNAG_03670 | Ire1 | Serine-threonine kinase and endoribonuclease | Confirmed |
| CNAG_05431 | Rim101 | pH-response transcription factor | Confirmed |
| CNAG_05650 | Ubp5 | Ubiquitin carboxyl-terminal hydrolase 7 | |

| | | | |
|---|---|---|---|
| **Transcription factor** | | | |
| CNAG_00151 | | Transcription elongation regulator 1 | Confirmed |
| CNAG_00252 | | Transcription elongation factor B, polypeptide 1 | Confirmed |
| CNAG_04630 | Yap2 | AP-1-like basic leucine zipper (bZIP) transcriptional activator | |
| CNAG_06156 | Fzc7 | Fungal specific transcription factor domain | |
| CNAG_06597 | Spt8 | Transcriptional activator SPT8 | |
| CNAG_05431 | Rim101 | pH-response transcription factor | Confirmed |
| CNAG_00193 | Gat1 | GATA type zinc finger protein asd-4 | |
| CNAG_00156 | Crz1 | Zinc finger transcription factor | Confirmed |
| CNAG_05222 | Nrg1 | Transcriptional regulator | Confirmed |
| CNAG_07464 | Mbs1 | Mbp1 and Swi4-like APSES protein 1 | Confirmed |
| CNAG_07435 | Hap2 | Putative transcriptional activator | |
| CNAG_01431 | Hob1 | Homeobox domain | |
| CNAG_01973 | Zfc2 | C2H2 zinc finger protein Zas1A | |
| CNAG_04263 | Bzp2 | Hypothetical protein; bZIP transcription factor | |
| CNAG_01173 | Lag1 | LAG1 family transcription factor, putative | |
| CNAG_04586 | Hob7 | LIM-homeobox protein | Confirmed |
| CNAG_01242 | HapX | CCAAT-binding transcription factor HapX | Confirmed |
| | | | |
| **Cell growth** | | | |
| CNAG_00570 | Pkr1 | cAMP-dependent protein kinase regulator | Confirmed |
| CNAG_02196 | Cdc11 | Septin | |
| CNAG_00293 | Ras1 | G-protein signaling GTPase | |
| CNAG_01918 | Bud6 | Cytoskeletal regulatory protein binding protein | |
| CNAG_07562 | Spa2 | Component of the polarisome | |
| CNAG_03622 | Tao3 | Cell polarity protein mor2 | |
| CNAG_06552 | Snf1 | AMP-activated serine/threonine-protein kinase catalytic subunit | Confirmed |
| CNAG_02727 | Hos4 | Similar to Avo2, Tor2 kinase component | Confirmed |
| CNAG_07499 | Chs8 | Chitin synthase | |
| CNAG_01431 | Hob1 | Homeobox domain | |
| CNAG_04263 | Bzp2 | Hypothetical protein; bZIP transcription factor | |

| | | | |
|---|---|---|---|
| | | | |
| **Nitrogen regulation** | | | |
| CNAG_02789 | | Nitrogen permease regulating protein | Confirmed |
| CNAG_00193 | Gat1 | GATA type zinc finger protein asd-4 | |
| | | | |
| **Glucose regulation** | | | |
| CNAG_06552 | Snf1 | AMP-activated serine/threonine-protein kinase catalytic subunit | Confirmed |
| CNAG_05222 | Nrg1 | Transcriptional regulator | Confirmed |
| CNAG_05410 | Vid24 | Glucose-induced degradation complex subunit | |
| CNAG_06327 | Mig1 | DNA-binding protein creA | Confirmed |
| | | | |
| **MFS/transporter** | | | |
| CNAG_06986 | Lpi8 | Sugar transporter | |
| CNAG_04536 | | Nicotinamide mononucleotide permease | Confirmed |
| CNAG_00574 | | Amino acid/metabolite permease | |
| | | | |
| **Glutamine family amino acid biosynthetic process** | | | |
| CNAG_06112 | Ura2/Cpa2 | Carbamoyl-phosphate synthase arginine-specific large chain | |
| CNAG_01238 | ArgJ | Arginine biosynthesis ArgJ, mitochondrial | |
| | | | |
| **Kynurenine** | | | |
| CNAG_04080 | | 3-hydroxyanthranilate 3,4-dioxygenase | Confirmed |
| CNAG_02780 | Bna5 | Kynureninase | Confirmed |
| | | | |
| **Miscellanious enzymes** | | | |
| CNAG_00472 | Pbr1 | Hypothetical protein; putative oxidoreductase | |
| CNAG_05477 | | Protein disulfide-isomerase | |
| CNAG_00542 | | Salicylate hydroxylase | |
| CNAG_03269 | | Aldehyde dehydrogenase | |
| | | | |
| **Hypothetical protein** | | | |
| CNAG_01050 | | Hypothetical protein | |
| CNAG_06189 | | Hypothetical protein | |

| | | | |
|---|---|---|---|
| CNAG_01069 | | Hypothetical protein | Confirmed |
| CNAG_00871 | Clr3 | Hypothetical protein | Confirmed |
| CNAG_03362 | | Hypothetical protein | |
| CNAG_00240 | | Hypothetical protein | |
| CNAG_01401 | | Hypothetical protein | |
| CNAG_06246 | | Hypothetical protein | |
| CNAG_00438 | | Hypothetical protein | |
| CNAG_02877 | Fzc51 | Hypothetical protein | Confirmed |
| CNAG_00458 | | Hypothetical protein | |
| CNAG_07772 | | Hypothetical protein | |
| CNAG_02533 | | Hypothetical protein | |
| CNAG_06595 | | Hypothetical protein | |
| CNAG_00137 | | Hypothetical protein | |
| CNAG_05159 | | Hypothetical protein | |
| CNAG_02788 | | Hypothetical protein | |
| CNAG_00520 | | Hypothetical protein | |
| CNAG_07488 | | Hypothetical protein | |
| CNAG_02975 | | Hypothetical protein | |
| CNAG_07564 | | Hypothetical protein | |
| CNAG_07703 | | Hypothetical protein | |
| CNAG_04810 | | Hypothetical protein | |
| CNAG_04751 | | Hypothetical protein | |
| CNAG_01694 | | Hypothetical protein | |
| CNAG_06499 | | Hypothetical protein | |
| CNAG_00543 | | Hypothetical protein | |
| CNAG_06820 | | Hypothetical protein | |
| CNAG_05512 | | Hypothetical protein | |

**Supplemental Table 3:** Mutants from the second screen with altered sensitivity to bortezomib and classified by biological function.

| **Biological Function** | **Protein Name** | **Description** | **log2 ratio** | Screen Overlap |
|---|---|---|---|---|
| **Cellular metabolic process** | | | | |
| CNAG_03355 | Tco4 | two-component-like sensor kinase | -0.110 | |
| CNAG_00452 | | isovaleryl-CoA dehydrogenase | 0.373 | |
| CNAG_03567 | Cbk1 | AGC/NDR/NDR protein kinase | 0.157 | |
| CNAG_06086 | Cdk8 | CMGC/CDK/CDK8 protein kinase | 1.461 | |
| CNAG_03139 | Cho2 | phosphatidylethanolamine N-methyltransferase | 0.102 | * |
| CNAG_03527 | Hel2 | cytoplasmic protein | -0.372 | |
| CNAG_05809 | Hem15 | ferrochelatase | 0.140 | |
| CNAG_06366 | Hrr2502 | protein kinase | -0.548 | |
| CNAG_00405 | Kic1 | STE/STE20/YSK protein kinase | 1.734 | |
| CNAG_04215 | Met3 | sulfate adenylyltransferase | -0.500 | |
| CNAG_04514 | Mpk1 | CMGC/MAPK protein kinase | 0.695 | |
| CNAG_00396 | Pka1 | AGC/PKA protein kinase | 0.693 | |
| CNAG_02820 | Pkh201 | serine/threonine protein kinase | 0.432 | |
| CNAG_04763 | Pmt2 | dolichyl-phosphate-mannose-protein mannosyltransferase | 0.573 | |
| CNAG_02048 | Put5 | proline dehydrogenase | -0.707 | |
| CNAG_01019 | Sod1 | superoxide dismutase [Cu-Zn] | -0.396 | |
| CNAG_01151 | Ubc5 | ubiquitin-conjugating enzyme E2 D/E | 0.384 | |
| CNAG_01720 | Vps23 | ESCRT-I complex subunit TSG101 | -0.360 | * |
| CNAG_02389 | Ypk101 | AGC protein kinase | -0.245 | |
| CNAG_00151 | | transcription elongation regulator 1 | -0.188 | * |
| CNAG_00263 | | serine/threonine/tyrosine-interacting protein | 0.444 | |
| CNAG_00397 | | 2-oxoisovalerate dehydrogenase E1 component, beta subunit | -0.199 | |
| CNAG_00537 | | carnitine O-acetyltransferase | -0.145 | |
| CNAG_00991 | | flap endonuclease 1 | 1.183 | * |

| | | | | |
|---|---|---|---|---|
| CNAG_01044 | | dihydroceramidase | 0.625 | |
| CNAG_01745 | | glycerol-3-phosphate dehydrogenase (NAD()) | 0.639 | |
| CNAG_02366 | | 4-aminobutyrate aminotransferase | 0.740 | |
| CNAG_02470 | | phosphoric monoester hydrolase | 0.383 | |
| CNAG_02662 | | cysteine protease ATG4 | 0.586 | |
| CNAG_03031 | | protein FRA10AC1 | -0.471 | |
| CNAG_03080 | | fatty acid elongase | -0.151 | * |
| CNAG_03269 | | aldehyde dehydrogenase | 2.673 | * |
| CNAG_03555 | | acylglycerone-phosphate reductase | 0.156 | |
| CNAG_03676 | | D-lactate dehydrogenase | -0.048 | |
| CNAG_03821 | | phosphatidylinositol 3-kinase | 1.337 | |
| CNAG_04493 | | ubiquitin carboxyl-terminal hydrolase 48, variant | 0.175 | |
| CNAG_04849 | | vacuolar protein | 0.354 | |
| CNAG_05070 | | sulfite reductase (NADPH) hemoprotein, beta-component | 0.311 | |
| CNAG_05820 | | tryptophan aminotransferase | -0.100 | |
| CNAG_06026 | | aspartate aminotransferase | -0.422 | |
| CNAG_06029 | | peptidyl-tRNA hydrolase ICT1 | -0.114 | |
| CNAG_06221 | | diphthamide biosynthesis protein 2 | -0.114 | |
| CNAG_06282 | | WD-repeat protein 48 | 0.625 | |
| CNAG_06497 | | microsomal epoxide hydrolase | -0.025 | |
| CNAG_06555 | | aromatic amino acid aminotransferase I | -0.126 | |
| CNAG_07629 | | endopolyphosphatase | 0.257 | |
| CNAG_07780 | | geranylgeranyl diphosphate synthase, type III | 0.649 | |
| **Transmembrane transport** | | | | |
| CNAG_00078 | | vacuolar protein | 0.674 | |
| CNAG_00235 | Amt1 | amt family ammonium transporter | -0.137 | |
| CNAG_00448 | | V-type H -transporting ATPase subunit AC39 | 0.241 | |
| CNAG_00560 | | V-type H -transporting ATPase subunit E | 0.491 | |
| CNAG_00823 | | cadmium ion transporter | 0.195 | |

| | | | | |
|---|---|---|---|---|
| CNAG_01118 | | AAT family amino acid transporter | 0.730 | |
| CNAG_01647 | | translocation protein SEC66 | 0.986 | |
| CNAG_01704 | Irk6 | serine/threonine protein kinase | 0.427 | |
| CNAG_01960 | | efflux protein EncT | 0.635 | |
| CNAG_03025 | | V-type H -transporting ATPase subunit D | -0.876 | |
| CNAG_03140 | | sugar transporter | 0.558 | |
| CNAG_03824 | | solute carrier family 25 (mitochondrial phosphate transporter), member 3 | 0.168 | |
| CNAG_03910 | Itr6 | D-xylose-proton symporter | 0.671 | |
| CNAG_04787 | | membrane protein | 0.079 | |
| CNAG_04982 | Fcy3 | cytosine-purine permease | 0.771 | |
| CNAG_05564 | | peroxin-10 | -0.666 | |
| CNAG_05674 | | solute carrier family 35, member E1 | 0.073 | |
| CNAG_05685 | | neutral amino acid transporter | -0.430 | |
| CNAG_05790 | | OPT family small oligopeptide transporter | -0.621 | |
| CNAG_05833 | | allantoate transporter | -0.218 | |
| CNAG_06536 | | monocarboxylic acid transporter | -0.222 | |
| CNAG_06538 | | monocarboxylic acid transporter | 0.390 | |
| CNAG_06610 | | MFS transporter | -0.162 | |
| **Cellular component organization or biogenesis** | | | | |
| CNAG_04806 | | golgi phosphoprotein 3 | 0.444 | |
| CNAG_00673 | | cytoplasmic protein | -0.814 | |
| CNAG_01536 | | myosin heavy chain | -0.263 | |
| CNAG_01583 | | vacuolar-sorting protein SNF7 | -0.103 | * |
| CNAG_02029 | Wsp1 | wiskott-Aldrich syndrome protein | -0.144 | |
| CNAG_03301 | Tim13 | mitochondrial import inner membrane translocase subunit TIM13 | 0.494 | |
| CNAG_03651 | | syntaxin 7 | 0.775 | |
| CNAG_04937 | | peroxin-12 | 0.324 | * |
| CNAG_05080 | | calcofluor white hypersensitive protein | -0.208 | |

| | | | | |
|---|---|---|---|---|
| CNAG_05875 | | cytochrome c heme-lyase | -0.244 | |
| CNAG_06376 | | vacuolar membrane protein | 1.439 | |
| CNAG_07347 | | heat shock protein | 0.900 | |
| CNAG_07810 | | mitochondrial protein | 0.644 | |
| **Regulation of DNA-templated transcription** | | | | |
| CNAG_01454 | Ste12 | transcription factor STE12 | -0.288 | |
| CNAG_03409 | Skn7 | osomolarity two-component system, response regulator SKN7 | 0.573 | |
| CNAG_03431 | Fzc48 | nuclear protein | -0.438 | |
| CNAG_04586 | Hob7 | LIM-homeobox protein | 0.728 | * |
| CNAG_05221 | H2A-4 | histone H2A.Z | 0.448 | |
| CNAG_07924 | Mcm1 | RNA polymerase II transcription factor | 0.427 | |
| **Cellular nitrogen compound metabolic process** | | | | |
| CNAG_00176 | | glutamate carboxypeptidase, variant | 0.072 | |
| CNAG_00328 | | DNA excision repair protein ERCC-5 | -0.218 | * |
| CNAG_00613 | Fcy1 | cytosine deaminase | 0.543 | |
| CNAG_01891 | | RAD57 protein | -0.203 | |
| CNAG_03898 | | phosphoadenosine phosphosulfate reductase | -0.660 | |
| CNAG_05642 | Fzc37 | nuclear protein | -0.298 | |
| CNAG_06371 | | guanine deaminase | 0.675 | |
| CNAG_06708 | | dCMP deaminase | 0.239 | |
| CNAG_06796 | | serine/arginine repetitive matrix protein 1 | 0.225 | |
| **Regulation of transcription by RNA polymerase II** | | | | |
| CNAG_02020 | | SWR1-complex protein 4 | 0.322 | |
| CNAG_02566 | Fkh2 | hepatocyte nuclear factor | 0.824 | |
| CNAG_03850 | Spt7 | transcriptional activator SPT7 | 0.629 | |
| CNAG_05392 | Zap104 | specific RNA polymerase II transcription factor | 0.858 | |

| | | | | |
|---|---|---|---|---|
| CNAG_05690 | Rpd304 | histone deacetylase RPD3 | -0.425 | |
| CNAG_07435 | Hap2 | transcription activator | 0.599 | * |
| CNAG_05431 | Rim101 | pH-response transcription factor pacC/RIM101 | 0.074 | * |
| CNAG_07724 | Cuf1 | ligand-regulated transcription factor | 0.776 | |
| CNAG_07725 | | HMG box factor | 0.628 | |
| **Carbohydrate metabolic process** | | | | |
| CNAG_00697 | Uge1 | UDP-glucose 4-epimerase | 0.532 | |
| CNAG_00914 | Kre6 | glucosidase | 0.154 | |
| CNAG_01257 | | aldo-keto reductase | 0.809 | |
| CNAG_02598 | Chi21 | chitinase | -0.578 | |
| CNAG_03916 | | glucose-6-phosphate isomerase | -1.256 | |
| CNAG_05148 | Cxt1 | beta-1,2-xylosyltransferase 1 | -0.061 | |
| CNAG_05653 | Mls1 | malate synthase A | 0.922 | |
| CNAG_07600 | | beta-glucosidase | 0.546 | |
| **Cellular aromatic compound metabolic process** | | | | |
| CNAG_02879 | | chloride channel protein, nucleotide-sensitive, 1A | 0.341 | |
| CNAG_00247 | Lys9 | alpha-aminoadipic semialdehyde synthase | -0.157 | |
| CNAG_02853 | | amidophosphoribosyltransferase | 0.294 | |
| CNAG_06172 | | transketolase | 0.970 | |
| CNAG_06645 | Mtd1 | methylenetetrahydrofolate dehydrogenase (NAD) | -0.174 | |
| CNAG_06692 | | glucosamine-phosphate N-acetyltransferase | 0.249 | |
| **Regulation of nucleic acid-templated transcription** | | | | |
| CNAG_00693 | | F-box and WD-40 domain-containing protein CDC4, variant | -1.399 | |
| CNAG_01948 | Fzc36 | nuclear protein | 0.793 | |
| **Cellular carbohydrate metabolic process** | | | | |

| | | | | |
|---|---|---|---|---|
| CNAG_01155 | Gut1 | glycerol kinase | 0.376 | |
| CNAG_03765 | Tps2 | trehalose-phosphatase | 0.055 | |
| CNAG_06977 | | L-iditol 2-dehydrogenase | 0.192 | |
| **Polysaccharide metabolic process** | | | | |
| CNAG_01230 | Mp98 | chitin deacetylase 2 | -0.288 | |
| CNAG_06031 | Kre63 | beta-glucan synthesis-associated protein, variant | 0.402 | |
| **Peptide biosynthetic process** | | | | |
| CNAG_04830 | | large subunit ribosomal protein L33 | -0.361 | |
| CNAG_04800 | | phenylalanine-tRNA ligase, alpha subunit | 0.729 | |
| CNAG_06754 | | RNA polymerase-associated protein CTR9 | 0.267 | |
| **Regulation of RNA biosynthetic process** | | | | |
| CNAG_02435 | Bwc2 | white collar 2 protein | 0.441 | |
| CNAG_07506 | Fap1 | transcriptional repressor NF-X1 | 1.326 | |
| **Miscellaneous** | | | | |
| CNAG_04093 | Yrm103 | putative transcription factor | -0.354 | |
| CNAG_02661 | Isp4 | identified spore protein 4 | 0.425 | |
| CNAG_00648 | | esterase/lipase | -0.829 | |
| CNAG_02373 | | ATP-binding protein | -0.460 | |
| CNAG_02989 | | PX domain-containing protein | -0.496 | |
| CNAG_03075 | | DNA polymerase delta subunit 3 | 0.448 | |
| CNAG_03202 | Cac1 | adenylate cyclase | 0.330 | |
| CNAG_03918 | Sog2 | ram signaling network protein | -0.177 | |
| CNAG_00740 | Snf5 | swi/snf chromatin-remodeling complex subunit | -0.158 | |
| CNAG_00005 | | TPR repeat-containing protein | -0.132 | |
| CNAG_00126 | | 2-deoxy-D-gluconate 3-dehydrogenase | 0.428 | |
| CNAG_00188 | | endoplasmic reticulum protein | -0.210 | |
| CNAG_00353 | Lpi16 | 3-oxo-5-alpha-steroid 4-dehydrogenase 1 | -0.170 | |

| | | | | |
|---|---|---|---|---|
| CNAG_00484 | | 2-oxoisovalerate dehydrogenase E2 component (dihydrolipoyl transacylase) | 0.271 | |
| CNAG_00542 | | salicylate hydroxylase | -0.261 | * |
| CNAG_00600 | Cap60 | capsular associated protein | -1.274 | * |
| CNAG_00641 | | transcription elongation factor SPT5 | 0.529 | |
| CNAG_00654 | Srx1 | sulfiredoxin | -0.469 | |
| CNAG_00662 | | carboxymethylenebutenolidase | -0.361 | |
| CNAG_00736 | Sec5 | exocyst protein, variant | -0.283 | |
| CNAG_00781 | | U6 snRNA-associated Sm-like protein LSm3 | 0.086 | |
| CNAG_00839 | | mitochondrial protein | 0.536 | |
| CNAG_00919 | | carboxypeptidase D | -0.348 | |
| CNAG_01095 | | BEM46 family protein | 0.214 | |
| CNAG_01108 | Dal2 | allantoicase | 0.677 | |
| CNAG_01507 | | protein CGI121 | -0.261 | |
| CNAG_01542 | | taurine catabolism dioxygenase TauD | 0.600 | |
| CNAG_01550 | | pre-rRNA-processing protein TSR3 | -0.097 | |
| CNAG_01584 | | hydrolase | 0.659 | |
| CNAG_01605 | | rossman fold oxidoreductase | -0.284 | |
| CNAG_01777 | | glyoxylate reductase | 0.606 | |
| CNAG_01798 | | phosphatase activator | 0.296 | |
| CNAG_01875 | | WD-repeat protein | 0.212 | |
| CNAG_02181 | | dihydrokaempferol 4-reductase | 0.641 | |
| CNAG_02217 | Chs7 | chitin synthase | -0.339 | |
| CNAG_02358 | | MRP family ATP-binding protein | 0.203 | |
| CNAG_02371 | | coiled-coil domain-containing protein 130 | 0.750 | |
| CNAG_02484 | | peptidyl-prolyl cis-trans isomerase, variant | 0.619 | |
| CNAG_02548 | | cobalamin synthesis protein, variant | -0.199 | |
| CNAG_02602 | | flavonol synthase | -0.548 | |
| CNAG_02670 | | bud emergence protein 1 | -0.497 | |
| CNAG_02885 | Cap64 | capsular associated protein | 1.258 | |

| | | | | |
|---|---|---|---|---|
| CNAG_02969 | | thioesterase, variant | 0.171 | |
| CNAG_02980 | | membrane dipeptidase | 0.469 | |
| CNAG_02990 | | nuclear protein | 0.501 | |
| CNAG_02998 | | nuclear protein | 0.481 | |
| CNAG_03065 | | ER-derived vesicles protein ERV14 | 0.456 | |
| CNAG_03149 | Liv9 | BRCA1-associated protein | -0.162 | |
| CNAG_03325 | Bch1 | ChAPs family protein | 0.020 | |
| CNAG_03388 | Rco1 | nuclear protein | 0.143 | |
| CNAG_03460 | | phosphoglycerate dehydrogenase | 0.307 | |
| CNAG_03523 | | solute carrier family 25, member 38, variant | -0.296 | |
| CNAG_03528 | | AP-2 complex subunit alpha | 0.188 | |
| CNAG_03576 | | cytochrome C assembly protein | 0.179 | |
| CNAG_03641 | | translation initiation factor 3 subunit D | 0.339 | |
| CNAG_03748 | | large subunit ribosomal protein L19 | -0.268 | |
| CNAG_03829 | | L-carnitine dehydratase/bile acid-inducible protein F | 0.294 | |
| CNAG_03949 | | 4-nitrophenyl phosphatase | -0.364 | |
| CNAG_04122 | | galactose dehydrogenase | -0.258 | |
| CNAG_04455 | | peroxisomal membrane protein 4 | -0.269 | |
| CNAG_04470 | | haloacid dehalogenase, type II | 0.558 | |
| CNAG_04520 | | D-aminoacylase | -0.111 | |
| CNAG_04642 | Tsp2 | tetraspanin Tsp2 | 0.831 | |
| CNAG_04659 | Pdc1 | pyruvate decarboxylase | -0.209 | |
| CNAG_04716 | | KH domain-containing protein | 0.282 | |
| CNAG_04835 | | dihydrodipicolinate synthase | -0.329 | |
| CNAG_04863 | Vps25 | ESCRT-II complex subunit VPS25 | -0.301 | * |
| CNAG_04895 | Fzc3 | nuclear protein | 0.932 | |
| CNAG_04904 | Chc1 | clathrin heavy chain | 1.213 | |
| CNAG_05201 | | DNA mismatch repair protein MSH4 | 0.762 | |
| CNAG_05369 | | beta-glucosidase, variant 1 | 0.800 | |

| | | | | |
|---|---|---|---|---|
| CNAG_05503 | | LIM domain-containing protein | 0.945 | |
| CNAG_05745 | | cytoplasmic protein | -0.064 | |
| CNAG_05765 | Ubc6 | ubiquitin-conjugating enzyme E2 J2 | -0.364 | |
| CNAG_05769 | | cytoplasmic protein | -0.446 | |
| CNAG_05818 | Chs5 | chitin synthase | 0.756 | |
| CNAG_05842 | | cytochrome P450 | 0.038 | |
| CNAG_05869 | | endopeptidase | 0.802 | |
| CNAG_06027 | | aryl-alcohol dehydrogenase | -0.503 | |
| CNAG_06154 | | cytoplasmic protein | 0.800 | |
| CNAG_06223 | Miz1 | E3 SUMO-protein ligase PIAS1 | -0.407 | |
| CNAG_06382 | | beta-transducin repeat containing protein | -0.537 | |
| CNAG_06392 | Sgf29 | SAGA-associated factor 29 | 0.338 | |
| CNAG_06439 | Alo1 | D-arabinono-1,4-lactone oxidase | 0.817 | |
| CNAG_06594 | | oxysterol binding protein | 0.095 | * |
| CNAG_06648 | Rtf1 | RNA polymerase-associated protein RTF1 | 0.081 | * |
| CNAG_06753 | | nuclear cap-binding protein subunit 1 | 0.260 | |
| CNAG_07029 | | vesicle-associated membrane protein 7 | -0.395 | |
| CNAG_07474 | | AP-2 complex subunit mu-1 | 0.711 | |
| CNAG_07483 | | DNA polymerase zeta subunit | -0.319 | |
| CNAG_07604 | Fre5 | metalloreductase | 0.911 | |
| CNAG_07622 | | COP9 signalosome complex subunit 1 | -0.143 | |
| CNAG_07636 | Csr2 | protoplast regeneration and killer toxin resistance protein | -1.113 | |
| CNAG_07712 | | NAD-dependent histone deacetylase SIR2 | 0.449 | |
| CNAG_04693 | Sin1 | target of rapamycin complex 2 subunit | 0.658 | |
| CNAG_05562 | Pbx2 | parallel beta-helix repeat protein | 0.942 | |
| CNAG_05590 | Tco2 | two-component-like sensor kinase | -0.223 | |
| CNAG_06464 | Liv7 | virulence related protein of unknown function | 0.882 | |
| CNAG_07534 | Trs130 | trafficking protein particle complex II-specific subunit 130 | 0.542 | |

| | | | | |
|---|---|---|---|---|
| CNAG_05791 | Zds3 | zds-like protein | 0.947 | |
| CNAG_07782 | | oxidoreductase | -0.577 | |
| **Hypothetical Protein** | | | | |
| CNAG_00698 | | hypothetical protein | -0.176 | |
| CNAG_02689 | | hypothetical protein | 0.600 | |
| CNAG_03058 | | hypothetical protein | -0.046 | |
| CNAG_03492 | | hypothetical protein | 3.381 | |
| CNAG_04638 | | hypothetical protein | 0.152 | |
| CNAG_07668 | | hypothetical protein | 0.209 | |
| CNAG_00033 | | hypothetical protein | 0.258 | |
| CNAG_00090 | | hypothetical protein | -0.400 | |
| CNAG_00127 | | hypothetical protein | 0.287 | |
| CNAG_00157 | | hypothetical protein | 0.633 | |
| CNAG_00177 | | hypothetical protein | 0.081 | |
| CNAG_00240 | | hypothetical protein | 0.718 | * |
| CNAG_00344 | | hypothetical protein | 0.263 | |
| CNAG_00374 | | hypothetical protein | -0.443 | |
| CNAG_00422 | | hypothetical protein | -0.187 | |
| CNAG_00465 | | hypothetical protein | 0.255 | |
| CNAG_00474 | | hypothetical protein | 0.410 | |
| CNAG_00503 | | hypothetical protein | -0.573 | |
| CNAG_00517 | | hypothetical protein | -0.241 | * |
| CNAG_00555 | | hypothetical protein | 0.483 | |
| CNAG_00583 | | hypothetical protein | 0.396 | |
| CNAG_00585 | | hypothetical protein | -1.022 | |
| CNAG_00626 | | hypothetical protein | 0.445 | |
| CNAG_00634 | | hypothetical protein | -0.183 | |
| CNAG_00647 | | hypothetical protein | 0.042 | |
| CNAG_00663 | | hypothetical protein | -0.307 | |

| | | | | |
|---|---|---|---|---|
| CNAG_00691 | | hypothetical protein | -0.216 | |
| CNAG_00715 | | hypothetical protein | -0.251 | |
| CNAG_00723 | | hypothetical protein | -0.691 | |
| CNAG_00759 | | hypothetical protein | 0.183 | |
| CNAG_00773 | | hypothetical protein | 0.666 | |
| CNAG_00794 | | hypothetical protein | 0.981 | |
| CNAG_00801 | | hypothetical protein | 0.083 | |
| CNAG_00915 | | hypothetical protein | 0.121 | |
| CNAG_00922 | | hypothetical protein | -0.333 | |
| CNAG_01004 | | hypothetical protein | 0.366 | |
| CNAG_01014 | Zfc4 | hypothetical protein | 0.364 | |
| CNAG_01020 | | hypothetical protein | 0.210 | |
| CNAG_01093 | | hypothetical protein | -0.277 | |
| CNAG_01207 | | hypothetical protein | 0.617 | |
| CNAG_01270 | | hypothetical protein | 0.224 | |
| CNAG_01275 | | hypothetical protein | 0.336 | |
| CNAG_01277 | | hypothetical protein | -0.070 | |
| CNAG_01297 | | hypothetical protein | 0.350 | |
| CNAG_01423 | | hypothetical protein | 0.518 | |
| CNAG_01476 | | hypothetical protein | 0.725 | |
| CNAG_01481 | | hypothetical protein | 0.063 | |
| CNAG_01525 | | hypothetical protein | 0.683 | |
| CNAG_01567 | | hypothetical protein | 0.340 | |
| CNAG_01698 | | hypothetical protein | 0.311 | |
| CNAG_01719 | | hypothetical protein | 0.163 | |
| CNAG_01738 | | hypothetical protein | 0.213 | |
| CNAG_01760 | | hypothetical protein | 0.146 | |
| CNAG_01825 | | hypothetical protein | 0.126 | |
| CNAG_01857 | | hypothetical protein | 0.255 | |

| | | | | |
|---|---|---|---|---|
| CNAG_01859 | | hypothetical protein | -0.164 | |
| CNAG_01989 | | hypothetical protein | -0.509 | |
| CNAG_02321 | | hypothetical protein | 0.335 | |
| CNAG_02332 | | hypothetical protein | 0.274 | |
| CNAG_02463 | | hypothetical protein | 0.325 | |
| CNAG_02582 | | hypothetical protein | -0.518 | |
| CNAG_02589 | | hypothetical protein | -0.046 | |
| CNAG_02594 | | hypothetical protein | 0.379 | |
| CNAG_02601 | | hypothetical protein | -0.428 | |
| CNAG_02606 | | hypothetical protein | -0.412 | |
| CNAG_02655 | | hypothetical protein | -0.271 | |
| CNAG_02659 | | hypothetical protein | -0.679 | |
| CNAG_02688 | | hypothetical protein | 0.374 | |
| CNAG_02822 | | hypothetical protein | -0.377 | |
| CNAG_02911 | | hypothetical protein | 0.862 | |
| CNAG_02942 | | hypothetical protein | -0.079 | |
| CNAG_03114 | | hypothetical protein | 0.822 | |
| CNAG_03118 | | hypothetical protein | -0.077 | |
| CNAG_03141 | | hypothetical protein | 0.126 | |
| CNAG_03169 | | hypothetical protein | 0.692 | |
| CNAG_03223 | | hypothetical protein | -0.366 | |
| CNAG_03324 | | hypothetical protein | 0.029 | |
| CNAG_03336 | Fzc50 | hypothetical protein | -0.166 | |
| CNAG_03529 | | hypothetical protein | -0.277 | |
| CNAG_03583 | | hypothetical protein | 0.275 | |
| CNAG_03667 | | hypothetical protein | -0.089 | |
| CNAG_03745 | | hypothetical protein | 0.752 | |
| CNAG_04059 | | hypothetical protein | 0.801 | |
| CNAG_04073 | | hypothetical protein | 0.257 | |

| | | | | |
|---|---|---|---|---|
| CNAG_04105 | | hypothetical protein | -0.466 | |
| CNAG_04185 | | hypothetical protein | 0.317 | |
| CNAG_04357 | | hypothetical protein | 0.129 | |
| CNAG_04502 | | hypothetical protein | 0.210 | |
| CNAG_04587 | | hypothetical protein | 0.635 | |
| CNAG_04634 | | hypothetical protein | -0.081 | |
| CNAG_04671 | | hypothetical protein | 0.141 | |
| CNAG_04875 | | hypothetical protein | 0.735 | |
| CNAG_04885 | | hypothetical protein | 0.849 | |
| CNAG_04891 | | hypothetical protein | 0.078 | |
| CNAG_04901 | | hypothetical protein | 0.168 | |
| CNAG_04992 | | hypothetical protein | 0.785 | |
| CNAG_05008 | | hypothetical protein | 0.164 | |
| CNAG_05022 | | hypothetical protein | 0.098 | |
| CNAG_05060 | | hypothetical protein | -0.335 | |
| CNAG_05074 | | hypothetical protein | 3.237 | |
| CNAG_05159 | | hypothetical protein | -0.317 | * |
| CNAG_05261 | | hypothetical protein | -0.316 | |
| CNAG_05262 | | hypothetical protein | 0.058 | |
| CNAG_05279 | | hypothetical protein | 0.153 | |
| CNAG_05295 | | hypothetical protein | -0.363 | |
| CNAG_05313 | | hypothetical protein | 0.901 | |
| CNAG_05325 | | hypothetical protein | -0.214 | |
| CNAG_05338 | | hypothetical protein | 0.569 | |
| CNAG_05463 | | hypothetical protein | -0.620 | |
| CNAG_05620 | | hypothetical protein | -0.432 | |
| CNAG_05668 | | hypothetical protein | 0.587 | |
| CNAG_05728 | | hypothetical protein | 0.829 | |
| CNAG_05774 | | hypothetical protein | -0.537 | |

| | | | | |
|---|---|---|---|---|
| CNAG_05789 | | hypothetical protein | 1.283 | |
| CNAG_06017 | | hypothetical protein | -0.205 | |
| CNAG_06213 | | hypothetical protein | -0.294 | |
| CNAG_06214 | | hypothetical protein | -0.066 | |
| CNAG_06216 | | hypothetical protein | -0.412 | |
| CNAG_06233 | | hypothetical protein | -0.130 | |
| CNAG_06234 | | hypothetical protein | 0.395 | |
| CNAG_06334 | | hypothetical protein | 0.764 | |
| CNAG_06516 | | hypothetical protein | -0.550 | |
| CNAG_06579 | | hypothetical protein | 0.488 | |
| CNAG_06604 | | hypothetical protein | 0.351 | |
| CNAG_06718 | | hypothetical protein | 0.377 | |
| CNAG_06727 | | hypothetical protein | 0.155 | |
| CNAG_06781 | | hypothetical protein | -0.218 | |
| CNAG_06805 | | hypothetical protein | -0.228 | |
| CNAG_06822 | | hypothetical protein | 0.264 | |
| CNAG_06904 | | hypothetical protein | -0.558 | |
| CNAG_06999 | | hypothetical protein | -0.246 | |
| CNAG_07306 | | hypothetical protein | 0.283 | |
| CNAG_07313 | | hypothetical protein | -0.430 | |
| CNAG_07322 | | hypothetical protein | 0.006 | |
| CNAG_07370 | | hypothetical protein | 0.709 | |
| CNAG_07385 | | hypothetical protein | -0.300 | |
| CNAG_07404 | | hypothetical protein | -0.600 | |
| CNAG_07420 | | hypothetical protein | 0.511 | |
| CNAG_07436 | | hypothetical protein | 0.643 | |
| CNAG_07452 | | hypothetical protein | -0.291 | |
| CNAG_07471 | | hypothetical protein | 0.460 | |
| CNAG_07517 | | hypothetical protein | 0.489 | |

| | | | | |
|---|---|---|---|---|
| CNAG_07523 | | hypothetical protein | -0.085 | |
| CNAG_07540 | | hypothetical protein | 0.735 | |
| CNAG_07543 | | hypothetical protein | 0.677 | |
| CNAG_07568 | | hypothetical protein | -0.242 | |
| CNAG_07578 | | hypothetical protein | -0.402 | |
| CNAG_07594 | | hypothetical protein | -0.411 | |
| CNAG_07596 | | hypothetical protein | -0.537 | |
| CNAG_07614 | | hypothetical protein | 0.154 | |
| CNAG_07631 | | hypothetical protein | 0.677 | |
| CNAG_07662 | | hypothetical protein | -0.195 | |
| CNAG_07691 | | hypothetical protein | -0.340 | |
| CNAG_07709 | | hypothetical protein | 0.785 | |
| CNAG_07727 | | hypothetical protein | 0.467 | |
| CNAG_07759 | | hypothetical protein | 0.190 | |
| CNAG_07762 | | hypothetical protein | -0.372 | |
| CNAG_07774 | | hypothetical protein | 0.400 | |
| CNAG_07775 | | hypothetical protein | -0.453 | |
| CNAG_07795 | | hypothetical protein | 0.343 | |
| CNAG_07833 | | hypothetical protein | 0.814 | |
| CNAG_07837 | | hypothetical protein | 0.855 | |
| CNAG_07845 | | hypothetical protein | 0.753 | |
| CNAG_07861 | | hypothetical protein | 1.213 | |
| CNAG_07881 | | hypothetical protein | 0.164 | |
| CNAG_07890 | | hypothetical protein | -0.438 | |
| CNAG_07931 | | hypothetical protein | -0.286 | |
| CNAG_07935 | | hypothetical protein | 0.772 | |

Figure S1. Confirmation of the growth defects of mutants sensitive to 25 µM of bortezomib at 24h and/or 48h at 37˚C. The analysis was performed as described in Figure 2C.

Figure S1

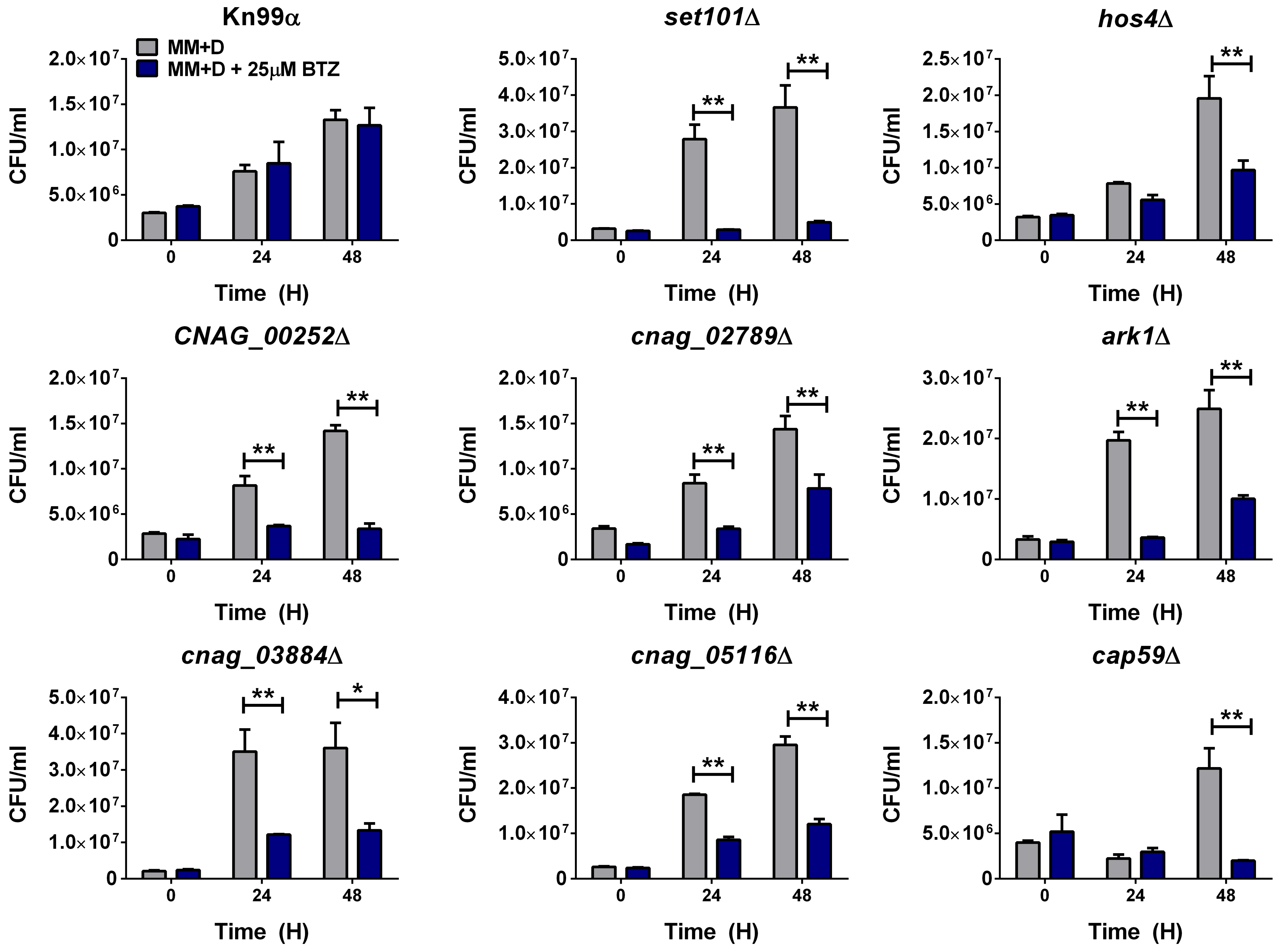

Kn99α
MM+D
MM+D + 25μM BTZ
set101Δ
hos4Δ
CNAG_00252Δ
cnag_02789Δ
ark1Δ
cnag_03884Δ
cnag_05116Δ
cap59Δ
CFU/ml
Time (H)
0
24
48
**
*

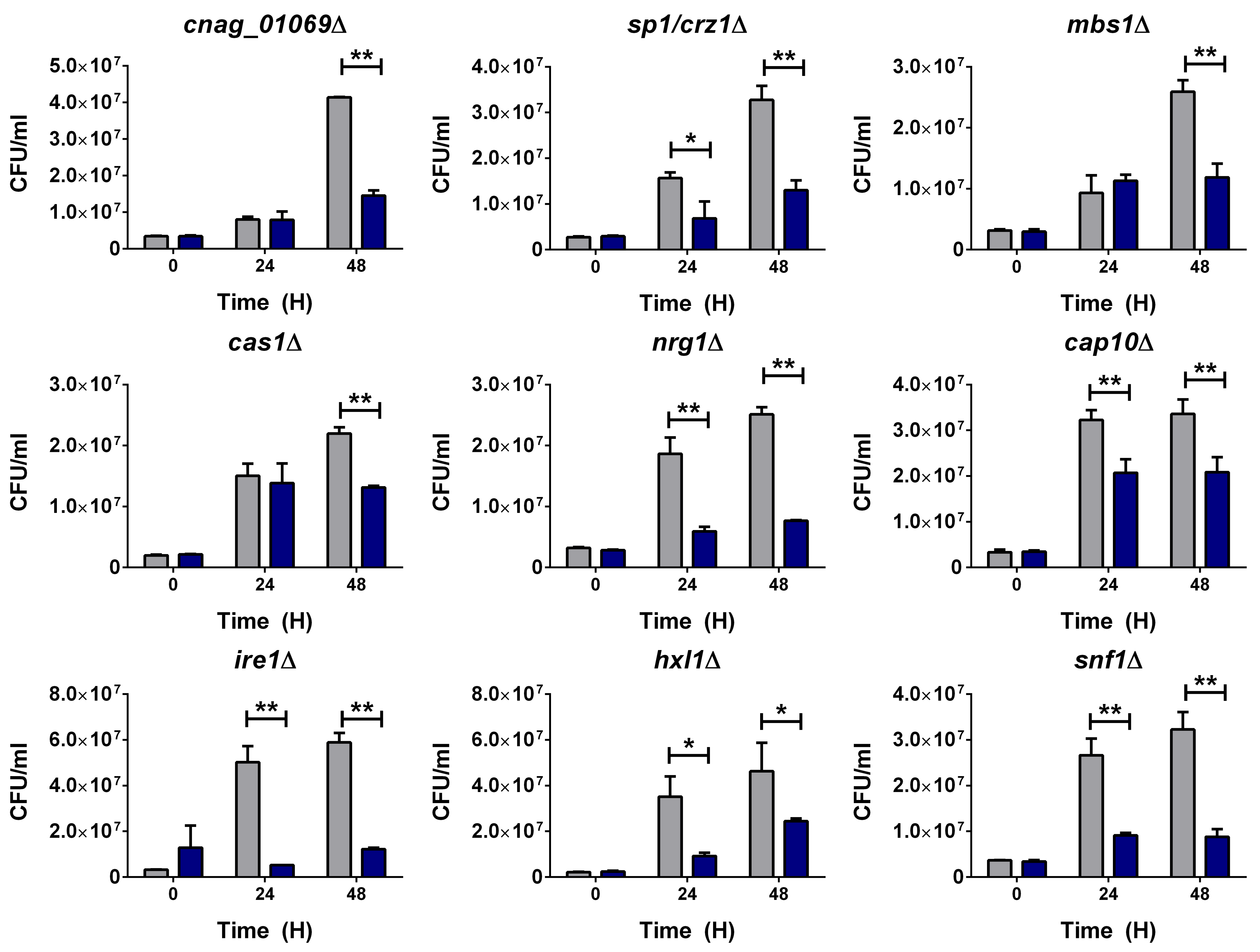

cnag_01069Δ
sp1/crz1Δ
mbs1Δ
cas1Δ
nrg1Δ
cap10Δ
ire1Δ
hxl1Δ
snf1Δ
CFU/ml
Time (H)
0
24
48
*
**

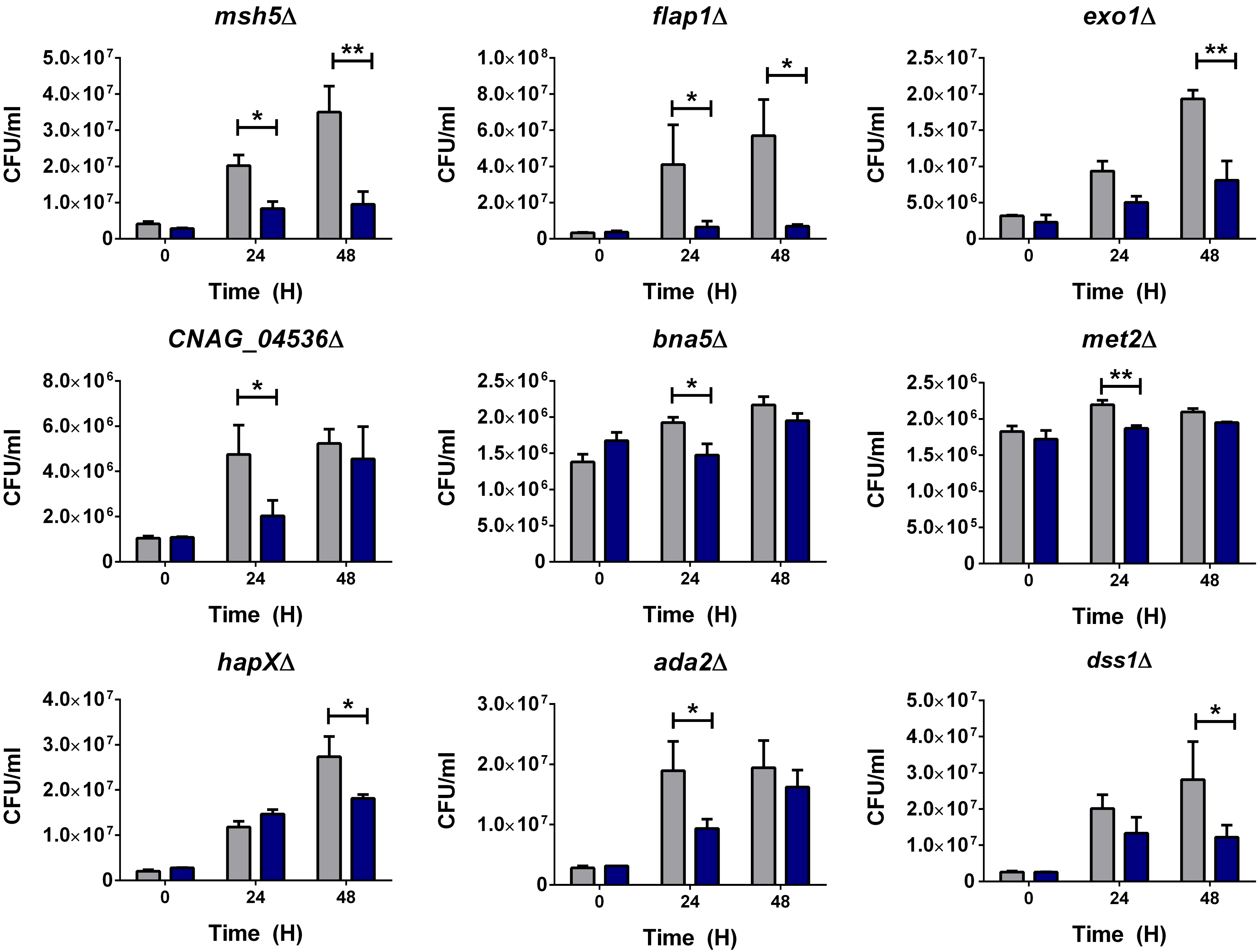

msh5Δ
flap1Δ
exo1Δ
CNAG_04536Δ
bna5Δ
met2Δ
hapXΔ
ada2Δ
dss1Δ
CFU/ml
Time (H)
0
24
48
*
**

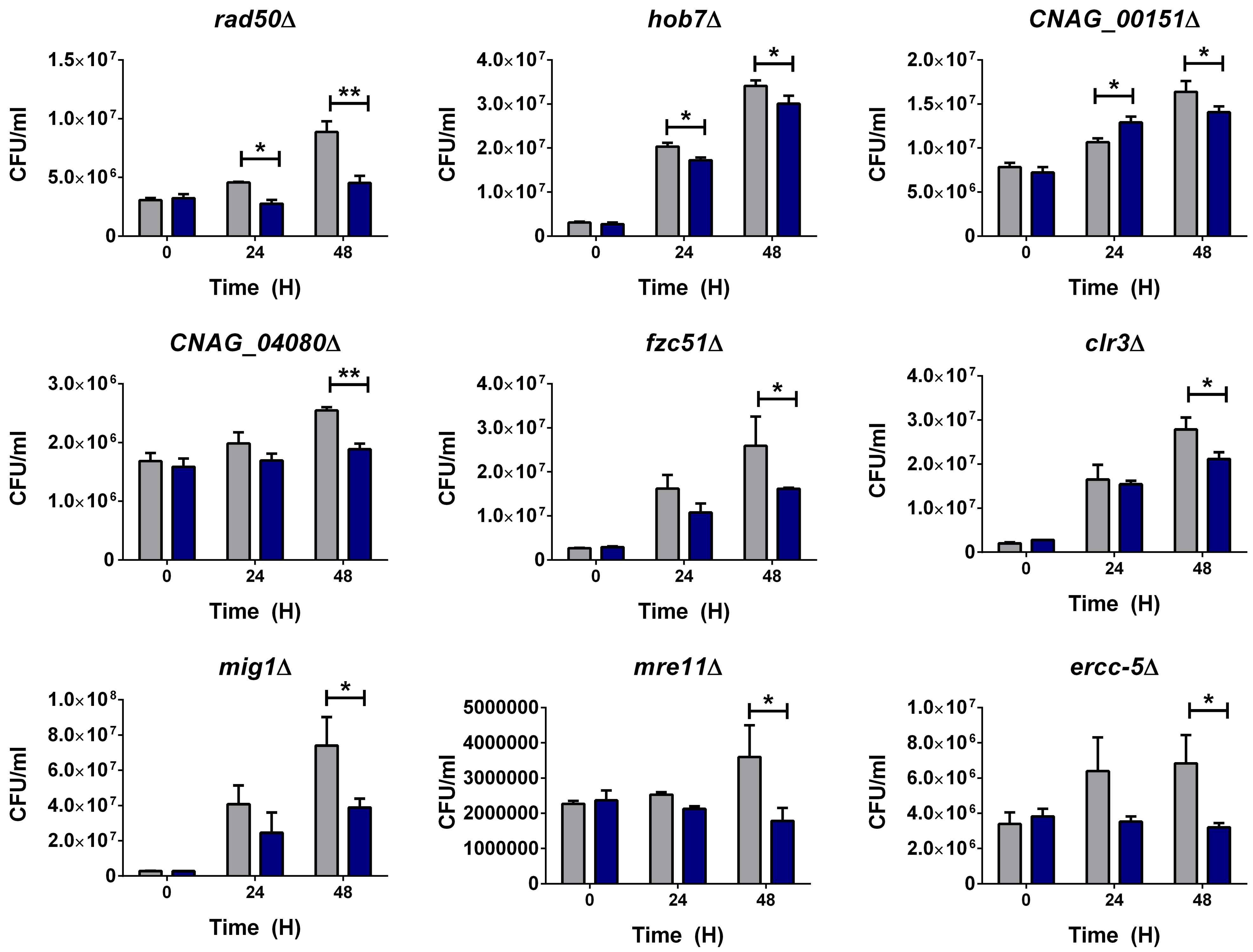

rad50Δ
hob7Δ
CNAG_00151Δ
CNAG_04080Δ
fzc51Δ
clr3Δ
mig1Δ
mre11Δ
ercc-5Δ
CFU/ml
Time (H)
0
24
48
1.5×10^7
1.0×10^7
5.0×10^6
4.0×10^7
3.0×10^7
2.0×10^7
1.0×10^7
2.0×10^7
1.5×10^7
3.0×10^6
2.0×10^6
1.0×10^6
1.0×10^8
8.0×10^7
6.0×10^7
4.0×10^7
5000000
4000000
3000000
2000000
1000000
1.0×10^7
8.0×10^6
6.0×10^6
4.0×10^6
2.0×10^6
*
**

**Figure S2. Evaluation of survival for infected mice treated with bortezomib, amphotericin B or both drugs.** The experiment was performed in parallel with the experiment in Fig. 5. Ten mice were used per treatment which started 24h post-infection and was performed intraperitoneally until day 8 with BTZ (1.4 mg/Kg at days 1, 4 and 8), AMB (0.5 mg/Kg/day), or BTZ+AMB (1.4 mg/Kg at days 1, 4 and 8 + 0.5 mg/Kg/day). Ordinary one-way ANOVA was used for statistical analyses and the values are presented in the table below the graph.

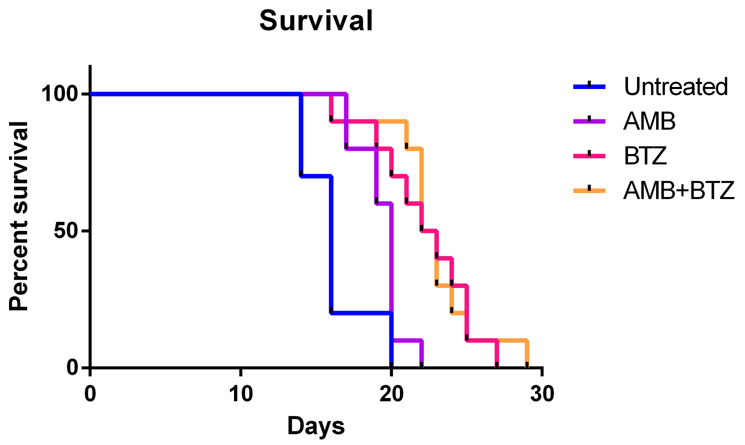


| Treatments | AMB | BTZ | AMB+BTZ |
|---|---|---|---|
| Untreated | **0.0091** | **0.0004** | **< 0.0001** |
| AMB | | **0.0118** | **0.0010** |
| BTZ | | | 0.7886 |
